# The Effects of Negative Affect and Dispositional Pessimism on Decision-Making Under Ambiguity

**DOI:** 10.5334/cpsy.167

**Published:** 2026-10-27

**Authors:** Molly Davidson, Vikki Neville, John Fennell, Elizabeth S. Paul, Iain D. Gilchrist, Michael Mendl

**Affiliations:** 1 Bristol Veterinary School, University of Bristol, Bristol, UK; 2 School of Psychological Science, University of Bristol, Bristol, UK

**Keywords:** Affective bias, anxiety, depression, pessimism, decision-making, drift-diffusion model

## Abstract

Negative affect has been shown, in both humans and non-human animals, to bias decision-making under ambiguity away from so-called ‘optimistic’ responses that can yield higher rewards but also risk greater losses. The judgement bias task, a cognitive task that uses ambiguous stimuli to measure such decisions as indicators of affective state, has successfully been translated from humans to animals and back again. However, questions remain regarding how exactly affect influences ‘optimistic’ or ‘pessimistic’ decisions, and the extent to which dispositional pessimism also influences these decisions. To further understand links between affect and decision-making, we carried out a judgement bias task in humans and measured self-reported affect and dispositional pessimism. In a pre-registered online experiment, participants completed questionnaires assessing anxiety, depression, and pessimism, and undertook a judgement bias task. Our results mirrored previous findings showing that self-reported negative affect is associated with negatively-biased decision-making under ambiguity; individuals who reported higher anxiety and depression made a significantly lower proportion of ‘optimistic’ responses to ambiguous stimuli in the judgement bias task. However, we did not find sufficient evidence to reject the null hypothesis that self-reported dispositional pessimism is associated with ‘pessimistic’ responses in the task. Drift-diffusion modelling revealed that the more negative (less ‘optimistic’) judgement bias associated with self-reported anxiety and depression, could be explained by a bias generated during the decision-making process, represented by a lower drift rate. But due to high comorbidity, we were unable to determine whether negative judgement bias is driven primarily by anxiety or depression.

## Introduction

Affective states play an important role in guiding decision-making, especially when there is ambiguity surrounding the decision outcome (Loewenstein & Lerner, 2003; Mendl & Paul, 2020). Judgement bias tasks capitalise on this relationship and have been successfully used to probe decision-making under ambiguity as an indicator of affective states in both human and non-human animals. These translational tasks were originally developed for use with animals, on the basis of findings from human psychology that people with mood disorders, or in induced negative affective states, interpreted ambiguous words, phrases, facial expressions, and future scenarios more negatively (e.g. Eysenck et al., 1991; Joormann & Gotlib, 2006; Mathews et al., 1989; Park et al., 2016; Wright & Bower, 1992). In Harding et al.’s (2004) original animal judgement bias task, rats were trained to press a lever when they heard a tone associated with a food reward, and to refrain from pressing the lever when they heard a different tone to avoid a punishment (white noise). Their responses to ambiguity were then tested by presenting tones of intermediate frequency. Rats that had been housed in unpredictable conditions designed to induce a negative affective state were slower to make lever press responses to intermediate ambiguous tones, and tended to make fewer such responses, indicating a reduced anticipation of reward and hence a ‘pessimistic’-like response to ambiguity.

Judgement bias tasks have now been used in over 200 studies as an indicator of animal affective state with results supporting the general hypothesis of an association between induced/presumed affective state and ‘optimism’ and ‘pessimism’ (see meta-analyses: Lagisz et al., 2020; Neville et al., 2020). They have also been successfully back-translated for use with human populations (e.g. Anderson et al., 2012; Aylward et al., 2020; Daniel-Watanabe et al., 2022; Iigaya et al., 2016; Neville et al., 2021a, 2021b; Paul et al., 2011; Schick et al., 2013). The human tasks can provide important insights into the processes underlying affective biases in judgements of ambiguity by using self-report as a marker of conscious affective experience. In this study, we therefore investigate how self-reported negative affective states (anxiety and depression) are associated with biases in decision-making within the judgement bias task.

We also ask whether self-reported dispositional ‘pessimism’ plays a role in biasing decisions that may be separable from affective state *per se*. Although judgement bias responses are typically described using the terms ‘optimism’ and ‘pessimism’ (or ‘optimistic-like’ and ‘pessimistic-like’), it is not clear to what extent dispositional pessimism might drive observed biases (Armbruster-Genç et al., 2024). Previous research has investigated the influence of affective state, finding for example that individuals reporting negative affect (especially using clinically relevant measures of depression and anxiety) are more likely to show ‘pessimistic’ decision-making under ambiguity (Aylward et al., 2020; Daniel-Watanabe et al., 2022). However, according to Beck’s cognitive model of depression (Beck et al., 1979), individuals with trait negative affect are more likely to have negative views about the future – i.e. increased pessimism – and therefore it is possible that predispositions to pessimistic thinking also influence decision-making under ambiguity. Here we investigate this issue by measuring both affect and dispositional pessimism in humans performing a judgement bias task. When referring to judgement bias task responses, we use the terms ‘optimistic’ and ‘pessimistic’ to maintain consistency with the literature, however they are used in an operational sense to describe task-specific response patterns rather than dispositional traits.

To analyse potential underlying processes, we employ computational modelling (see Neville et al., 2024), applying a drift-diffusion model (DDM: Ratcliff et al., 2016) which combines choice and reaction time data to provide insight into the mechanisms underlying the decision-process, typically in two-alternative forced choice tasks. The DDM conceptualises decisions as a process of evidence accumulation toward one of two decision thresholds, each representing an alternative choice. Decisions are modelled as the rate of accumulation of evidence driving towards one or other threshold, combined with other parameters including whether the start point of the evidence accumulation process is closer to one of the thresholds.

One question which drift-diffusion modelling can be used to address is whether a bias towards one response is more likely to occur due to a pre-existing (start point) bias towards one option prior to stimulus presentation, or due to a difference in the rate of evidence accumulation (drift rate) during the decision-making process. Consider data which show a negative judgement bias i.e. there is a higher proportion of ‘pessimistic’ responses than ‘optimistic’ responses. If this was generated by an *a priori* bias, it would be reflected in a DDM by a relative start point parameter value of under 0.5 (with start point = 0.5 reflecting no *a priori* bias) due to the participant making the ‘pessimistic’ response more quickly and more often. Conversely, if the participant had similar reaction time profiles for both responses but responded ‘pessimistically’ more often, this would be reflected by a negative drift rate (with drift rate = 0 reflecting no drift towards either boundary) and suggest that the negative bias was generated *during* the decision-making process.

It is possible to hypothesise that the influences of affective and dispositional pessimism on judgement bias may be related to a ‘start point’ bias within a DDM framework; both pessimistic individuals and those experiencing a negative mood state may be more likely to anticipate negative outcomes prior to stimulus presentation, and this would be reflected in a negatively biased start point. However, there is some evidence from previous studies that negative affect is associated with a negative drift rate in human judgement bias task studies (Aylward et al., 2020; Daniel-Watanabe et al., 2022), so we investigate this issue further here.

The judgement bias task we employ is similar to that used in previous studies (e.g. Anderson et al., 2012; Aylward et al., 2020; Daniel-Watanabe et al., 2022; Iigaya et al., 2016; Neville et al., 2021a; Schick et al., 2013). For example, Aylward et al. (2020) used a two-tone discrimination task in which participants had to press one key when a certain tone played to receive a high reward of £4 and had to press another key when a different tone played to receive a low reward of £1. Pressing the wrong key in each case yielded no reward. During testing, participants were also presented with an intermediate ambiguous tone on some trials, and their responses to this ambiguous tone were investigated. Participants with mood or anxiety disorder symptoms made more ‘pessimistic’ responses, i.e. selected the key predicting the low reward indicating a negatively-biased anticipation of the smaller reward rather than the larger reward, compared to asymptomatic control participants. Daniel-Watanabe et al. (2022) extended these findings in a similar task using an individual differences approach; higher self-reported depression and anxiety symptom scores were associated with a more negative judgement bias, confirming that a relationship between negative affect and decision-making can be detected across individuals.

In this study, we aimed to replicate the finding that self-reported negative affect negatively biases decision-making under ambiguity in humans (Daniel-Watanabe et al., 2022), whilst also exploring the relationship between dispositional pessimism and biased decisions. To explore the overall replicability, robustness and generality of findings, we used a different variant of the judgement bias task, different anxiety and depression questionnaires, and a different type of drift-diffusion modelling approach to Daniel-Watanabe et al. (2022).

To achieve these aims, we ran a large-scale pre-registered online study that allowed us to look at individual differences in self-reported anxiety (GAD-7 score: Spitzer et al., 2006) and depression (PHQ-8 score: Kroenke et al., 2001; Wu et al., 2020). The GAD-7 and PHQ-8 questionnaires ask participants to report on symptoms occurring during the preceding two weeks, and we assumed that these provided a reliable metric of their general affective state when participating in the study. We also assessed dispositional pessimism using the Revised Life Orientation Test (LOT-R: Scheier et al., 1994), as well as responses in a high/low reward active choice judgement bias task. In the task, participants had to decide whether to make the ‘optimistic’ response (the response associated with receiving a high reward) or the ‘pessimistic’ response (the response associated with receiving a low reward) when shown ambiguous visual stimuli. We hypothesised that there would be negative associations between each of the questionnaire measures (self-reported anxiety, depression, and dispositional pessimism) and ‘optimistic’ decision-making under ambiguity. We also applied drift-diffusion modelling to investigate whether negative judgement biases were associated with a reduced start point parameter, indicating an *a priori* negative bias, and/or whether they were associated with a more negative drift-rate during the decision-making process itself.

## Methods

### Ethics statement

Ethical approval was granted by the University of Bristol Human Research Ethics Committee (Ref: 2020-7982-7929). Participants provided informed consent via an online consent form.

### Preregistration

This experiment was preregistered on the OSF on the 28th of June 2021: https://osf.io/shvfr. All preregistered analyses are reported, and exploratory analyses are labelled as such.

### Participant recruitment

The experiment was run online using Gorilla (Anwyl-Irvine et al., 2020, www.gorilla.sc/). Participants were recruited and reimbursed for their time via Prolific (www.prolific.com). The inclusion criteria were: (1) individuals over 18 years old, (2) individuals who currently lived in the UK, and (3) individuals who selected ‘United Kingdom’ as their nationality on Prolific. Data were collected in 11 batches between the 16th of July and the 10th of September 2021. Prior to data collection, the whole experiment was piloted with 10 participants who were not included in the reported data set. Participants who completed the study were paid £1.80 for their time plus a 20p bonus. To encourage engagement with the task, participants were told that they had a chance to earn the 20p bonus depending on whether they responded correctly on one trial selected at random. In fact, all participants received the 20p bonus regardless of performance. To ensure that participants were attending to the possible trial outcomes, they were also told that for every 100 people that completed the task, the best performing participant would receive an extra bonus of £10. This bonus was paid out accordingly.

### Sample size

To determine the target sample size, we used G*Power (v3.1.9.7, Faul et al., 2007) to conduct a power calculation. The power calculation was based on a finding from a similar study (Daniel-Watanabe et al., 2022), where a significant correlation of r = –0.13 was observed between the proportion of ‘optimistic’ responses to ambiguous stimuli in a decision-making task and Beck Depression Inventory (BDI) scores. The calculation used p < 0.05 and 90% power giving a target sample size of 503 participants. We recruited 540 participants in total, based on a 93% completion rate observed in Daniel-Watanabe et al. (2022). 20 participants withdrew from the study, and data from a further 4 participants were excluded due to poor performance in the task (responding incorrectly on 50% or more of either of the unambiguous stimuli trials). This gave a final sample size of 516 (273 female; 236 male; 4 other; 3 prefer not to say; aged 18–73; median age = 36 years). Due to the low number of participants reporting their sex as ‘other’ or ‘prefer not to say’, statistical comparisons are only made between participants reporting their sex as ‘male’ or ‘female’. Data from participants reporting their sex as ‘other’ or ‘prefer not to say’ are still included in the analyses.

### Questionnaires

Participants completed the Generalised Anxiety Disorder Assessment (GAD-7) (Spitzer et al., 2006) to measure anxiety and the Patient Health Questionnaire (PHQ-8) (Kroenke et al., 2001; Wu et al., 2020) to measure depression. The GAD-7 and PHQ-8 were selected as they ask about how individuals have felt over the last two weeks which we used to indicate current negative affective states. They are also clinically relevant, being routinely used as clinical measures of the severity of anxiety and depression symptoms in the UK (NHS England, 2025). Participants also completed the Revised Life Orientation Test (LOT-R) (Scheier et al., 1994), a validated psychological tool used to measure individual differences in optimism and pessimism. LOT-R scores were reverse coded to produce a pessimism (LOT-RP) score for each participant. Participants’ age in years, sex, and handedness were also recorded in a demographics questionnaire. All participants completed the study in a fixed order: consent form, judgement bias task (see below), LOT-R questionnaire, PHQ-8 questionnaire, GAD-7 questionnaire, demographics questionnaire.

### Judgement bias task

Participants were instructed to ensure that they were sat upright, 60 cm from the screen and that their screen was level. To ensure that the stimuli appeared correctly, participants were required to calibrate their screen using Gorilla’s screen calibration function. The stimuli used were symmetrical Gabor patches (10 cm x 10 cm, contrast: 50%, standard deviation of the Gaussian envelope: 1.5 degrees, grating spatial frequency: 0.5 cycles/degree) of various orientations, inspired by the stimuli used by Iigaya et al. (2016).

The task required participants to classify the Gabor patches as tilted to the left or right of vertical by making keyboard press responses: ‘Z’ for left, ‘M’ for right (Figure 1). A trial was initiated by pressing the spacebar, after which a fixation cross was displayed in the centre of the screen for 500 ms, followed by the stimulus for 250 ms. Participants could respond from the beginning of stimulus presentation and were instructed to respond “as quickly and as accurately as possible”. Feedback (£0, +£1, or +£4) was given immediately and displayed for 500 ms.

**Figure 1:**
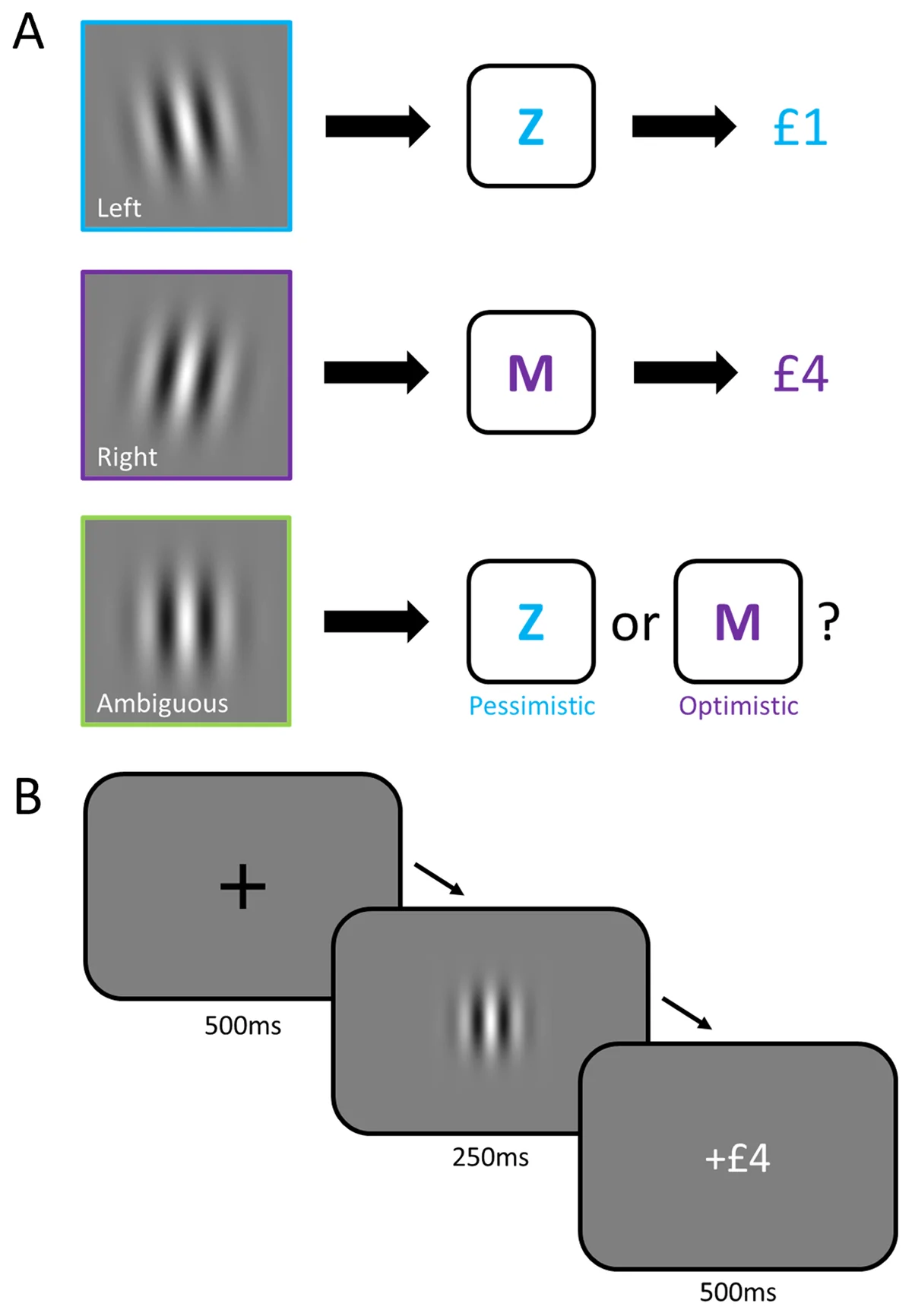
Judgement bias task schematic. **A)** Participants were required to classify Gabor patches as tilted to the left or right of vertical. In this example, if the patch was tilted left they should press ‘Z’ to nominally receive a £1 reward whereas if the patch was tilted right they should press ‘M’ to nominally receive a £4 reward. If they made the incorrect response they received £0. During testing participants were also presented with ambiguous patches which were only slightly tilted to the left or right. For ambiguous trials pressing ‘Z’ was classed as a ‘pessimistic’ response and pressing ‘M’ as an ‘optimistic’ response. **B)** A trial was initiated by a spacebar press. A fixation cross was displayed in the centre of the screen for 500 ms followed by the stimulus for 250 ms. Participants made a keyboard press response (‘Z’ or ‘M’) and were shown feedback for 500 ms.

The task used two unambiguous stimuli (–3.2 and 3.2 degrees from vertical) and two ambiguous stimuli (–0.05 and 0.05 degrees from vertical); the (un)ambiguity of these stimuli was previously validated by Iigaya et al. (2016). Correct responses were nominally rewarded with either a high reward (£4) or low reward (£1) depending on the Gabor patch orientation (left or right) and participants received £0 for an incorrect response. Participants were told that the aim of the task was to earn as much money as possible. The amount of ‘money’ won in the task determined which 6 participants received the £10 bonuses.

The high/low reward direction was counterbalanced within participants to account for any directional bias i.e. where a participant is predisposed to select either the left or right key, regardless of the stimulus. Participants therefore completed two testing blocks, the first where left-tilted patches were nominally rewarded with £4 and the second where left-tilted patches were rewarded with £1 (and vice versa for right-tilted patches). Both testing blocks consisted of 120 trials with all four stimuli equally represented across testing trials in a fixed random order. Prior to each testing block, participants completed a training block of 20 trials using only the unambiguous stimuli. Only data from the testing blocks were included in the analysis.

The key outcome of interest from the judgement bias task was the proportion of ‘optimistic’ responses to the ambiguous stimuli. Here we define an ‘optimistic’ response as the response associated with the high-reward (£4) direction. Responses to the two ambiguous stimuli (Ambiguous Negative and Ambiguous Positive) were compared using a paired-samples t-test, the assumptions of which were met; a Shapiro–Wilk test indicated that the difference scores did not deviate from normality (W = 0.998, p = 0.777) supported by visual inspection of the histogram and Q-Q plots. A statistically significant difference was observed (t(515) = –2.15, p = 0.032) albeit with a very small effect size (mean difference = –0.008; Cohen’s dz = –0.09, 95% CI [–0.18, –0.01]). No significant difference in ‘optimistic’ responses between the Ambiguous Negative and Ambiguous Positive stimuli was found in post-hoc comparisons of our trial-level analysis (z = 2.496, p = 0.055; see supplementary material). Based on this, and existing evidence that Gabor patch stimuli of these orientations cannot be reliably characterised above chance levels (Iigaya et al., 2016), the proportion of ‘optimistic’ responses were combined for both ambiguous stimuli to give each participant an ‘Optimistic Response’ score.

### Statistical analyses

Analyses were run in R (version 4.3.3, R Core Team, 2021). General linear model (GLM) assumptions were verified by visual inspection of model residuals. For the whole dataset, data from trials where the reaction times were very short (<200 ms) were excluded from the analysis as these were assumed to be accidental key presses rather than deliberate responses (1321/145,600 or 0.91% excluded) (Myers et al., 2022). Similarly, data from trials where reaction times were too long (>2000 ms), and therefore not likely to relate to the presented stimuli, were excluded from the analysis (844/145,600 or 0.58% excluded) (Myers et al., 2022). In addition, data from 4 participants were excluded due to poor performance in the task (responding incorrectly on 50% or more of either of the unambiguous stimuli trials). Mean accuracy for the remaining participants on negative unambiguous trials was 93% (1SD = 0.09) and on positive unambiguous trials was 97% (1SD = 0.05). The median number of ambiguous trials following the exclusion of trials with reaction times <200 ms and >200 ms was 120 (range: 59–120).

As per our preregistered plan, to determine whether demographic and questionnaire variables were significantly associated with the proportion of ‘optimistic’ responses to ambiguous stimuli in the judgement bias task (Optimistic Responses), we used a GLM containing the following independent variables: age, sex, anxiety score (from the GAD-7), depression score (from the PHQ-8) and pessimism score (from the LOT-RP). Likelihood ratio tests were used to assess the significance of these variables. To investigate the effects of sex on Optimistic Responses, *post-hoc* comparisons were conducted using a simultaneous pairwise Tukey procedure for general linear hypotheses with the R package ‘multcomp’ (Hothorn et al., 2008). In our preregistration we also anticipated that the three questionnaire scores would be highly correlated and therefore would require separate GLMs to independently test for associations between each questionnaire score and Optimistic Responses. Pearson’s correlations were used to assess the association between the three questionnaire scores. As these were highly correlated with each other as expected, we ran the three further GLMs, each containing one of the questionnaires as well as age and sex. Model fit was compared using Akaike Information Criterion (AIC). The Optimistic Responses summary score was used to make our analysis comparable to ‘standard’ analyses of human and animal judgement bias tasks, however recognising the loss of information with this approach, additional trial-level analyses were performed to check whether similar results were observed from the complete dataset (see supplementary material).

### Drift-diffusion modelling

To investigate how negative affect influences the decision-making process at the individual level, drift-diffusion modelling (DDM) was applied to the judgement bias task data using the Python toolbox HDDM (version 1.0.1 with Python 3.9.13, Wiecki et al., 2013). Only data for the ambiguous judgement bias task trials were included in the model. The model was fitted hierarchically using Markov chain Monte Carlo (MCMC) sampling and contained 4 parameters: drift rate (*v*), threshold separation (*a*), start point bias (*z*), and non-decision time (*t*). We ran 10,000 MCMC iterations, discarding the first 500 samples as burn-in. Starting values were optimised prior to sampling to facilitate convergence. Model convergence was assessed through visual inspection of trace plots and autocorrelation functions. These diagnostics indicated stable post-burn-in sampling, good mixing, and no evidence of systematic trends. In addition, posterior predictive checks indicated that the fitted model adequately reproduced the observed reaction time distributions, suggesting satisfactory convergence and model fit. Parameter recovery checks showed good recovery, and a hold-out validation procedure confirmed good predictive performance for held-out data (see supplementary material for details).

DDM parameters were estimated using a fully hierarchical Bayesian model implemented in HDDM, such that individual estimates were regularised by group-level priors. Although HDDM allows covariates to be included directly, initial analysis revealed that anxiety, depression, and pessimism questionnaire scores were correlated, making their simultaneous inclusion prone to multicollinearity and reduced identifiability. Instead, relationships between DDM parameters and demographic and questionnaire variables were subsequently examined in separate general linear models (GLMs). For each DDM parameter we ran a GLM containing age and sex, and three further GLMs containing age, sex, and one of the questionnaire scores: anxiety (GAD-7) score, depression (PHQ-8) score, or pessimism (LOT-RP) score. For each DDM parameter, the model with the best fit was selected using the lowest AIC value and results for this model only are reported. To investigate the effects of sex on the DDM parameter estimates, post-hoc comparisons were conducted using a simultaneous pairwise Tukey procedure for general linear hypotheses with the R package ‘multcomp’ (Hothorn et al., 2008). Additionally, a Pearson’s correlation was used to assess the association between Optimistic Responses in the judgement bias task and drift rate. All analyses relating to drift-diffusion modelling were not preregistered and are purely exploratory.

## Results

### Judgement bias

The mean proportion of ‘optimistic’ responses to the negative unambiguous stimulus was 0.07 (1SD = 0.09) and to the positive unambiguous stimuli was 0.97 (1SD = 0.05) (Figure 2). The mean proportions of ‘optimistic responses to the two ambiguous stimuli, Ambiguous Negative and Ambiguous Positive, were 0.59 (1SD = 0.14) and 0.60 (1SD = 0.14) respectively (Figure 2). For subsequent analyses, ambiguous responses were combined for each participant to give the proportion of ‘optimistic’ responses to ambiguous stimuli in the judgement bias task (Optimistic Responses); the mean Optimistic Responses value was 0.60 (1SD = 0.13).

**Figure 2:**
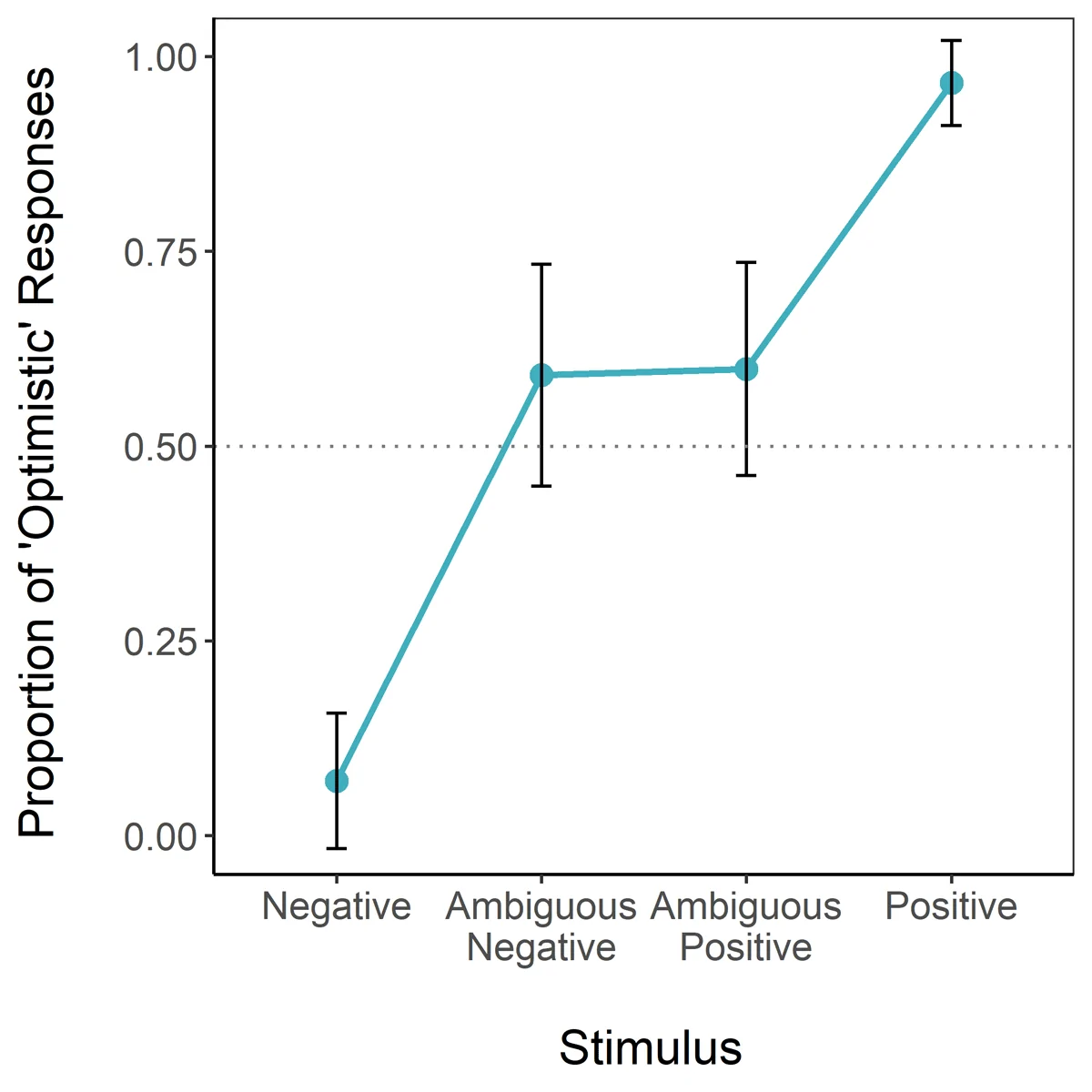
Mean Proportion of ‘Optimistic’ Responses for each stimulus ± 1 SD. Each participant completed 240 trials in total with the four stimuli equally represented. n = 516.

The initial GLM containing age, sex, anxiety, depression, and pessimism revealed significant effects of age and sex on Optimistic Responses, but no significant effect of any of the three questionnaire scores. Optimistic Responses significantly decreased with age (β ± SE = –0.001 ± < 0.001, LRT = 9.352, p = 0.002, Figure 3). Sex was a significant predictor of Optimistic Responses (LRT = 36.474, p < 0.001); post-hoc analysis revealed that male participants had significantly higher Optimistic Responses than females (male mean: 0.63, female mean: 0.56; z = 5.961, p < 0.001, Figure 4). The effects of anxiety (LRT = 1.031, p = 0.310), depression (LRT = 0.493, p = 0.483), and pessimism (LRT = 0.153, p = 0.696) on Optimistic Responses were not significant in this model. However, as the 3 questionnaires scores were highly correlated (particularly anxiety and depression: r(514) = .81, p < 0.001, Figure 5), we also ran separate GLMs for each of the questionnaires which controlled for sex and age. There was no significant effect of pessimism score on Optimistic Responses (LRT = 1.095, p = 0.295) however both anxiety (β ± SE = –0.003 ± 0.001, LRT = 5.962, p = 0.015) and depression (β ± SE = –0.003 ± 0.001, LRT = 5.465, p = 0.019) scores were significantly negatively associated with Optimistic Responses (Figure 5). Model fit was similar for the anxiety and depression models (AICs = –654.675 and –654.178, respectively). Findings from the trial-level analyses were consistent with those from the main analyses; there was no evidence of a significant effect of pessimism score on task responses, however both anxiety and depression scores were significantly negatively associated with the number of ‘optimistic’ responses made in the judgement bias task (see supplementary material).

**Figure 3:**
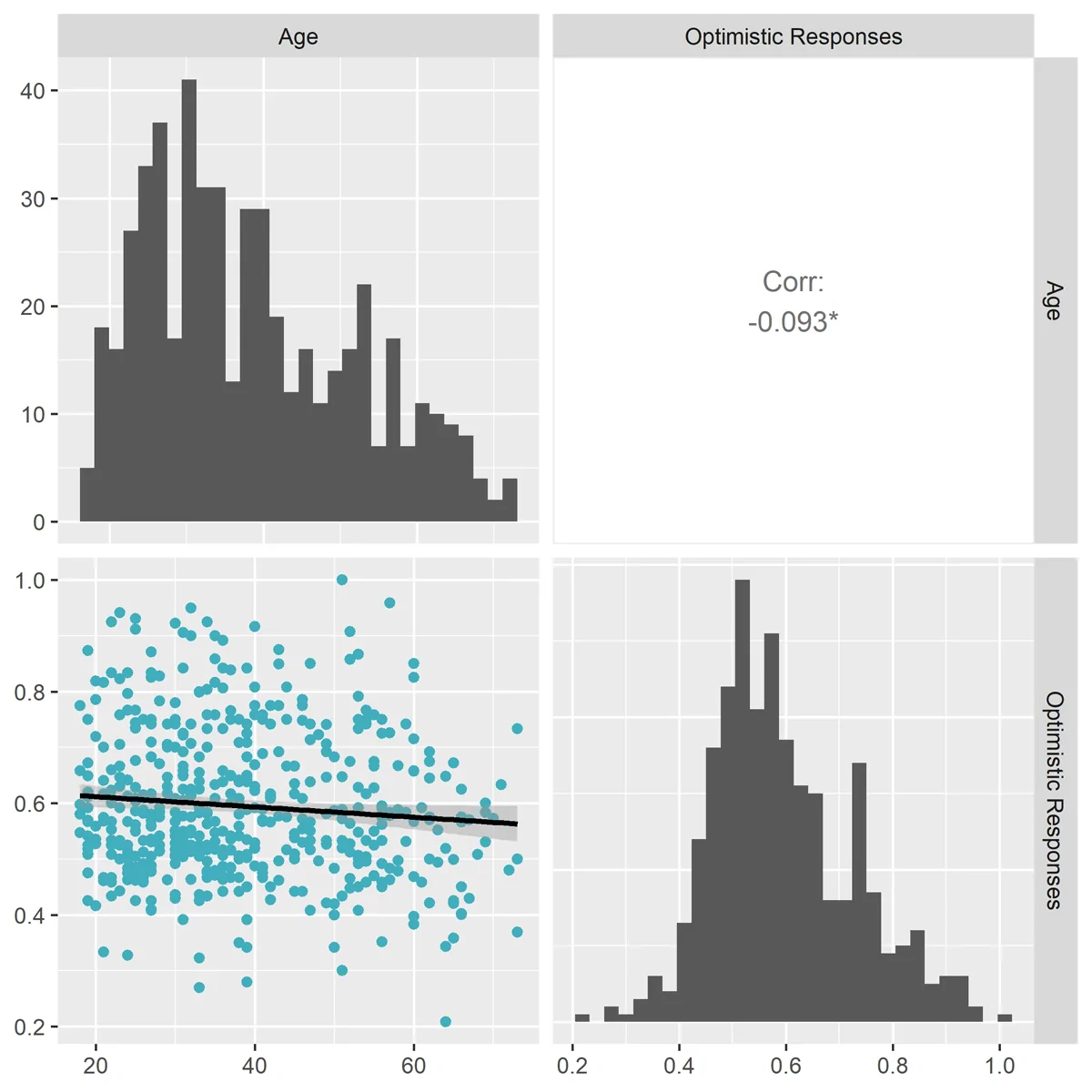
Association between Optimistic Responses and Age. Diagonally from top left to bottom right: frequency distributions for age and Optimistic Responses. Lower left: scatterplot with linear model lines ± shaded 95% confidence intervals. Upper right: Pearson correlation coefficient *p < 0.05). n = 516.

**Figure 4:**
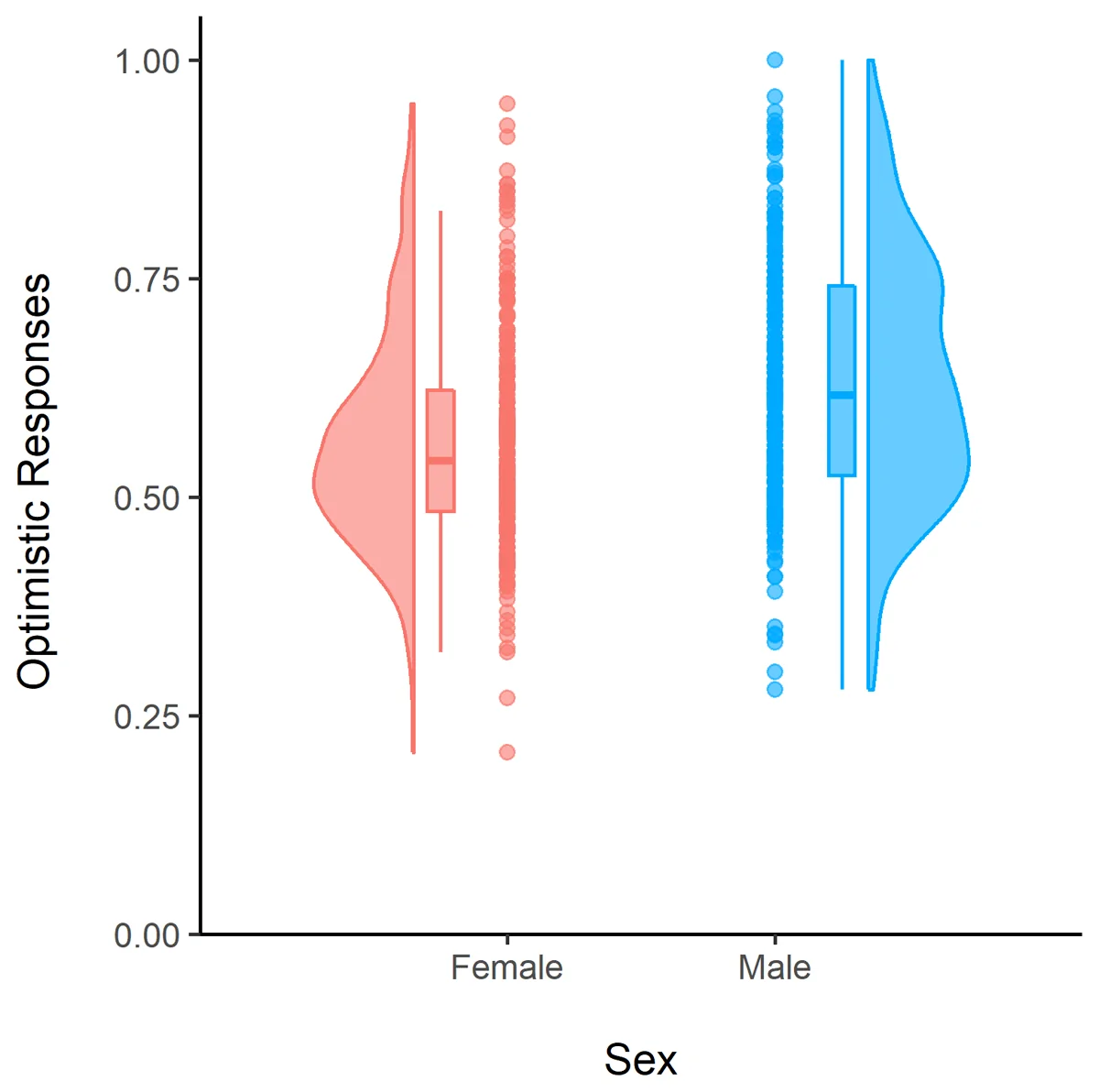
Optimistic Responses by Sex. Data are shown for females (n = 273, red) and males (n = 236, blue).

**Figure 5:**
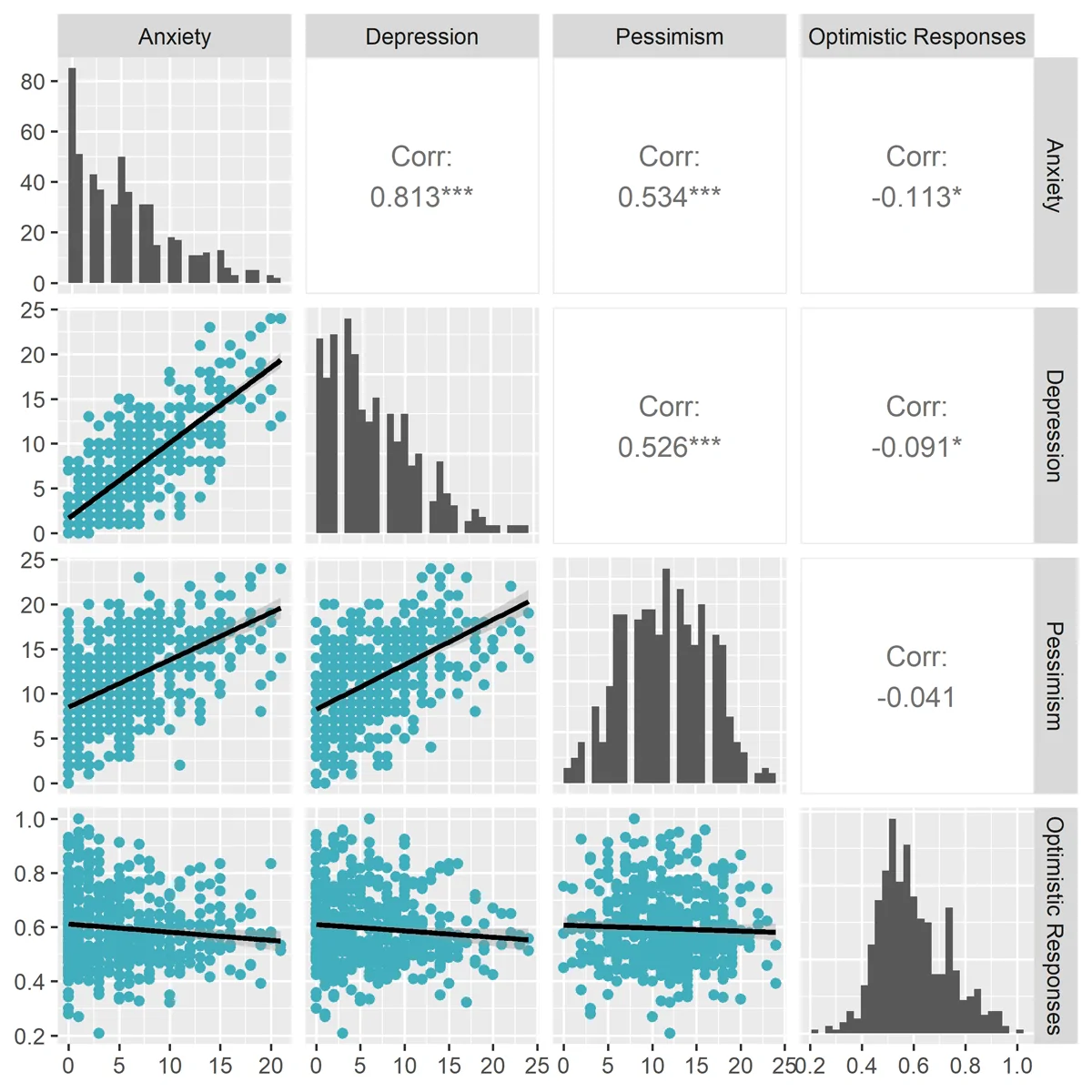
Associations between Anxiety, Depression, Pessimism, and Optimistic Responses Diagonally from top left to bottom right: frequency distributions for each variable. Lower left portion: scatterplots for each pair of variables with linear model lines ± shaded 95% confidence intervals. Upper right portion: Pearson correlation coefficients for each pair of variables (***p < 0.001, *p < 0.05). n = 516.

### Drift-diffusion modelling

Drift-diffusion model parameters were estimated for each participant. For each of the four DDM parameters (drift rate, threshold separation, start point bias, and non-decision time) we ran a GLM containing age and sex, and three further GLMs containing age, sex, and one of the questionnaire scores: anxiety, depression, or pessimism. The model with the best fit was selected using the lowest AIC value and the results for these models are reported here (Table 1).

**Table 1:** Model comparison for general linear models (GLMs) pairing drift-diffusion model (DDM) parameters with questionnaire predictors. Akaike Information Criterion (AIC) values were used to compare model fit. The GLM with the lowest AIC value for each DDM parameter is highlighted and results for this model are reported.


DDM PARAMETER	QUESTIONNAIRE	AIC

Drift rate (*v*)	None	738.127

	**Anxiety**	**730.891**

	Depression	732.203

	Pessimism	738.741

Threshold separation (*a*)	None	–322.323

	Anxiety	–322.484

	**Depression**	**–324.343**

	Pessimism	–320.483

Start point bias (*z*)	**None**	**–2270.476**

	Anxiety	–2268.561

	Depression	–2268.694

	Pessimism	–2269.109

Non-decision time (*t*)	**None**	**–859.795**

	Anxiety	–857.912

	Depression	–857.867

	Pessimism	–857.801


#### Drift rate (*v*)

For the drift rate parameter, the model containing anxiety score had the lowest AIC (Table 1). Drift rate significantly decreased with higher anxiety scores (β ± SE = –0.014 ± 0.004, LRT = 9.236, p = 0.002, Figure 6), and with age (β ± SE = –0.005 ± 0.002, LRT = 9.093, p = 0.003). Sex was also a significant predictor of drift rate (LRT = 31.364, p < 0.001); post-hoc analysis revealed that male participants had significantly higher drift rates than females (z = 5.515, p < 0.001). Optimistic Responses and drift rate were very strongly positively correlated (r(514) = .96, p < 0.001, Figure S1).

**Figure 6:**
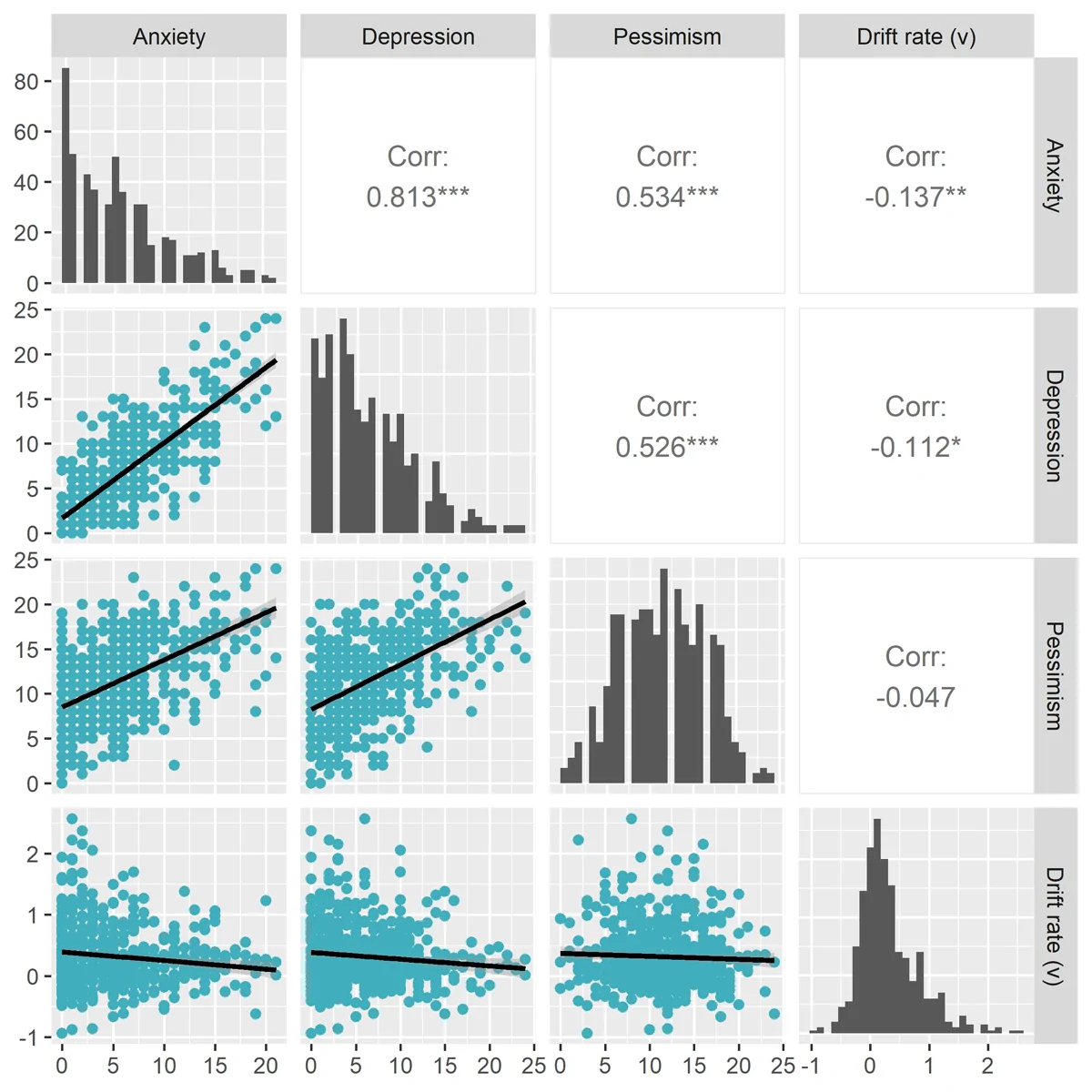
Associations between Anxiety, Depression, Pessimism, and Drift rate (*v*). Diagonally from top left to bottom right: frequency distributions for each variable. Lower left portion: scatterplots for each pair of variables with linear model lines ± shaded 95% confidence intervals. Upper right portion: Pearson correlation coefficients for each pair of variables (***p < 0.001, **p < 0.01, *p < 0.05). n = 516.

#### Threshold separation (*a*)

For the threshold separation parameter, the model containing depression score had the lowest AIC (Table 1). Threshold separation showed a marginal positive association with depression scores (β ± SE = 0.003 ± 0.002, LRT = 4.020, p = 0.045, Figure S2). Threshold separation also significantly increased with age (β ± SE = 0.002 ± 0.001, LRT = 9.474, p = 0.002). There was no significant effect of sex on threshold separation (LRT = 2.013, p = 0.570).

#### Start point bias (*z*)

For the start point bias parameter, the model containing no questionnaire score had the lowest AIC (Table 1). There was no significant effect of age (LRT = 0.248, p = 0.619) or sex (LRT = 4.213, p = 0.239) on start point bias.

#### Non-decision time (*t*)

For the non-decision time parameter, the model containing no questionnaire score had the lowest AIC (Table 1). Non-decision time significantly increased with age (β ± SE = 0.004 ± < 0.001, LRT = 124.083, p < 0.001). There was no significant effect of sex (LRT = 2.921, p = 0.404) on non-decision time.

## Discussion

We conducted a human judgement bias study to investigate how self-reported affect and dispositional pessimism may bias decision-making under ambiguity. Using a large-scale pre-registered online experiment where participants completed questionnaires assessing anxiety, depression, and pessimism, and undertook a judgement bias task, we confirmed that self-reported negative affect is associated with negative judgement biases during decision-making under ambiguity; individuals who reported higher anxiety (GAD-7) and depression (PHQ-8) scores made a significantly lower proportion of ‘optimistic’ responses when faced with ambiguous stimuli in the judgement bias task. This is consistent with previous findings in the literature (e.g. Aylward et al., 2020; Daniel-Watanabe et al., 2022), despite differences in the judgement bias tasks and questionnaires used, suggesting that the association between negative affect and ‘pessimistic’ decision-making under ambiguity is robust and generalised. This result is also consistent with translational judgement bias studies conducted with non-human animals, where animals presumed to be in more negative affective states tend to show negative (less ‘optimistic’) biases in judgement bias tasks (Lagisz et al., 2020; Neville et al., 2020).

We also showed that while self-reported dispositional pessimism (LOT-RP) scores are significantly positively correlated with both anxiety and depression scores, we did not find sufficient evidence to reject the null hypothesis that self-reported pessimism is inversely associated with ‘optimistic’ responses in the judgement bias task. This is contrary to our original hypothesis and suggests that the LOT-R questionnaire and the judgement bias task may be capturing different components or constructs of affect. A recent study by Armbruster-Genç et al. (2024) also found no significant correlation between LOT-R scores and judgement bias task responses. If we take the LOT-R questionnaire as a gold standard measure of dispositional optimism (or inversely, pessimism), these findings call into question whether judgement bias task responses should be referred to as ‘optimistic’ or ‘pessimistic’, or whether a change in terminology would be more accurate, such as ‘positively biased’ and ‘negatively biased’ responses. This is particularly relevant for experiments involving non-human animals where individuals are often classified as being ‘optimistic’ or ‘pessimistic’ based on judgement bias task outputs (e.g. Espigares et al., 2022; Lecorps et al., 2018; Papciak et al., 2013; Rygula et al., 2012); although such terms can be defined operationally (e.g. Matheson et al., 2008), their use may misleadingly imply human-like constructs. In the present paper, we continue to use the terms ‘optimistic’ and ‘pessimistic’ to remain consistent with the wider literature, however they are used operationally to describe task responses rather than broader personality traits.

Computational modelling of decision-making data allows us to gain a richer understanding of how affect could be influencing underlying decision-making processes. Here, we used the drift-diffusion model (Ratcliff et al., 2016) to understand the potential cognitive mechanisms underlying the bias towards the ‘pessimistic’ response or away from the ‘optimistic’ response observed in those with higher anxiety and depression scores. All drift-diffusion model analyses were not preregistered meaning any findings are exploratory and should be interpreted cautiously. Despite our theory-based prediction that individuals reporting higher anxiety and depression scores would have a pre-existing bias towards making the ‘pessimistic’ response to ambiguous stimuli (Beck et al., 1979), the results suggest that the negative bias emerged during the decision-making process rather than *a priori*; anxiety scores were significantly associated with a decreased drift rate but not with a change in start point bias. In fact, the proportion of ‘optimistic’ responses to ambiguous stimuli and drift rate were highly and significantly correlated. This is consistent with previous findings in humans (Aylward et al., 2020; Daniel-Watanabe et al., 2022) and rats (Hales et al., 2016).

We also found a marginal positive association between depression scores and threshold separation values, a parameter which represents how much information is required to reach a decision. In previous human judgement bias studies, no relationship between negative affect and threshold separation has been observed (Aylward et al., 2020; Daniel-Watanabe et al., 2022). However, rats showing a negative bias (induced by a pharmacological manipulation or chronic stress) have also been found to have an increased threshold separation compared to controls (Hales et al., 2016). Similarly, studies using different decision-making tasks to investigate mood disorders have also identified a relationship between more negative affect and increased threshold separation values using drift-diffusion modelling (Edgar et al., 2025; Lawlor et al., 2020). This could potentially reflect more cautious decision-making with increased depression severity.

It is important to note that many other tasks have been developed to explore affective biases in decision-making. For example, the judgement bias task used here shares similarities with the probabilistic reward task (PRT) developed by Pizzagalli and colleagues, which has been widely used to study anhedonia and depression (Pizzagalli et al., 2008a; Pizzagalli et al., 2008b). In contrast to our high/low reward design, the PRT uses two stimuli reinforced with equal reward magnitude but differing probabilities, such that correct identification of one (‘rich’) stimulus is rewarded more frequently than the other (‘lean’) stimulus, leading healthy participants to develop a response bias towards the rich stimulus; this bias is typically reduced in individuals with anhedonia and depression (Pizzagalli et al., 2008a; Pizzagalli et al., 2008b). Drift-diffusion modelling of PRT performance has shown that this reduced response bias is associated with both reduced drift rate and reduced starting point bias (Shen et al., 2024). In contrast to judgement bias tasks, which rely on explicitly learned reward contingencies before testing responses to ambiguity, the PRT captures the gradual development of a response bias under uncertainty, requiring participants to learn reward probabilities over time (Pizzagalli et al., 2008a; Pizzagalli et al., 2008b). While some PRT studies link negative affect to altered learning rate, there is evidence to suggest that these effects instead reflect reduced reward sensitivity (Huys et al., 2013). In this case, the observed effects of negative affect on decision-making in both tasks may be driven by overlapping underlying mechanisms. The use of multiple complementary tasks such as these in the same participants, coupled with computational modelling of the data, would be beneficial in further characterising affective biases.

A secondary aim of this study was to explore whether anxiety or depression symptoms drive ‘pessimistic’ decision-making in the judgement bias task. We found anxiety and depression scores to be very highly correlated. This meant that they could not be included in the same model, and when considered separately, both anxiety and depression scores were significantly associated with a negative judgement bias. Given the high multicollinearity between anxiety and depression measures, it is not possible to determine which is the primary driver of our observed results. Accordingly, we suggest that the findings indicate negative affect more broadly influences ‘pessimistic’ decision-making under ambiguity. When analysed separately, anxiety and depression scores were also both significantly associated with a lower drift rate. Again, we observed only a small difference in model fit, so conclude that the lower drift rate is associated with negative affect, rather than anxiety or depression specifically. Daniel-Watanabe et al.’s (2022) study found that depression scores, measured using the Beck Depression Inventory II (BDI: Beck et al., 1996), were more strongly related to judgement bias task performance than anxiety scores, measured using the State-Trait Anxiety Inventory (STAI: Spielberger et al., 1983). However, their exploratory factor analysis suggested that depression and anxiety scores should be combined into a single latent factor. Due to high comorbidity of anxiety and depression, other studies have also combined anxiety and depression (e.g. Aylward et al. (2020) compared a mood disorder group to a control group). Whilst it is possible that negative judgement bias is driven by a negative trait factor common to both anxiety and depression, future research could use more specific questionnaires, such as measures based on the tripartite model (e.g. the Mood and Anxiety Symptom Questionnaire; MASQ; Watson et al., 1995), to better dissociate anxiety and depression symptoms and explore whether particular symptoms or sets of symptoms are more strongly associated with judgement bias.

Studies such as ours which have used an individual differences approach by sampling from the general population have also found very small effect sizes (e.g. Daniel-Watanabe et al., 2022; Armbruster-Genç et al., 2024) compared to studies which have compared patient and control groups (e.g. Aylward et al., 2020), potentially suggesting that the observed negative bias may be best characterised as a clinical mood disorder symptom; using symptom score cutoffs (GAD-7 ≥ 10; PHQ-8 ≥ 10) only 21% of participants in our study would be classed as having moderate to severe anxiety and 26% as having moderate to severe depression (Spitzer et al., 2006; Kroenke et al., 2001; Wu et al., 2020). This may also help to explain why the normally distributed responses we received to the LOT-RP pessimism questionnaire were not significantly related to judgement bias task responses.

One concern highlighted by the small effect sizes observed here and in other human judgement bias studies (e.g. Daniel-Watanabe et al., 2022; Armbruster-Genç et al., 2024), is how sensitive and selective these tasks are to detecting and measuring negative (or positive) affect. Currently it is unclear whether small or null effects in these studies reflect limited sensitivity or specificity of the judgement bias tasks themselves, limitations of the self-report measures used to capture affect, or both. These human back-translated judgement bias task studies are relatively new therefore further work is needed to disentangle these possibilities and evaluate their reliability and validity for translational research (Pike et al., 2021). If the relationship between affect and judgement bias is genuinely weak, even in humans who can explicitly report their affective state (albeit imperfectly), this necessarily limits how strongly judgement bias outcomes can be interpreted in animals, where affect must be inferred indirectly. In addition, animal studies are likely to be underpowered, particularly when weaker affect manipulations are used, as it is difficult to achieve large enough sample sizes to detect small effects. Importantly, however, meta-analyses of animal judgement bias studies indicate that both pharmacological and non-pharmacological affect manipulations produce small to small-to-moderate effects in the predicted direction (Lagisz et al., 2020; Neville et al., 2020). Taken together, these findings suggest that judgement bias is sensitive to affective manipulations, but potentially with limited precision, reinforcing the need to interpret it as one component within a broader framework of cognitive, behavioural, and physiological measures of affect.

It is also important to consider that responses in judgement bias tasks may be influenced by other factors and to measure and control for these where possible. When piloting the task, we found that many participants showed a directional bias towards one of the two decision keys (Z or M), which we addressed by using within subject counterbalancing by swapping the high/low reward direction halfway through the task. We also found significant effects of age and sex on judgement bias. Consistent with Daniel-Watanabe et al.’s, (2022) study, the proportion of ‘optimistic’ responses made to ambiguous stimuli in the task significantly decreased with age. Male participants were also significantly more ‘optimistic’ than females in the task, a finding not observed in Daniel-Watanabe et al.’s (2022) study. Whilst these age and sex effects are interesting, they were not a focus of the study and were not preregistered therefore they should be treated as exploratory findings that warrant further investigation, especially as the existing literature on this topic is limited. It is also likely that judgement bias tasks are capturing other non-affect related traits, such as risk-taking behaviour which has been shown to be higher in males than females (e.g. Byrnes et al., 1999; Charness & Gneezy, 2012). Due to time constraints, we did not include an IQ measure, however previous work suggests that IQ has a significant effect on these types of tasks where logic may to some extent override emotional biases (Daniel-Watanabe et al., 2022). In addition, the types of reinforcers used within the judgement bias task are likely to influence responses. For example, we might predict that tasks which use high versus low rewards would be more sensitive to changes in reward processing and therefore more relevant to depression symptoms (Whitton et al., 2015) whereas a task using a reward versus punishment design may be more sensitive to changes in punishment processing and therefore more relevant to anxiety symptoms (Robinson et al., 2013).

While our study focused on the relationship between self-reported anxiety and depression during the past two weeks and judgement bias performance, responses may also have been influenced by shorter-term changes in participants’ affective states (Loewenstein & Lerner, 2003). Over the short test session, we expected that participants’ moods would be relatively stable and therefore would predictably influence task and questionnaire responses over the whole session. However, due to the fixed study order it is possible that task performance could have influenced subsequent questionnaire responses. For example, poorer performance on the judgement bias task may have induced a temporarily more negative mood, potentially inflating self-reported symptoms of anxiety or depression. A fixed study order was used to facilitate the investigation of individual differences, and the judgement bias task was completed first as we believed it likely that explicitly directing participants’ attention to their affective state during self-report could attenuate the extent to which affect influenced decision-making in the judgement bias task (Kassam & Mendes, 2013). Alternatively, completing questionnaires assessing anxiety, depression, and pessimism may induce a negative affective state which could influence task performance. Nonetheless, potential order effects should be considered when interpreting our findings.

Shorter-term (event-triggered) emotions may last only seconds to minutes and are also likely to vary over a test-session, particularly in response to trial feedback e.g. winning £4 may induce a positively-valenced emotion which could positively bias decision-making on a subsequent trial (Neville et al., 2021a). Future experiments could include self-reported measures of both current mood and short-term emotion to differentiate between their effects on judgement bias task responses; a questionnaire to assess immediate mood (e.g. the Profile of Mood States (POMS) questionnaire (Grove & Prapavessis, 1992)) could be included alongside the anxiety and depression questionnaires used in this study and moment-to-moment affective states could also be assessed using the addition of affect grids throughout the task which allow participants to report on their current affective valence and arousal (Neville et al., 2021a, 2021b). In addition to affective states influencing task responses, it is likely that affective states also influenced how participants responded to the questionnaires which we used to capture negative affect over the past two weeks. To ensure that the questionnaires used here are capturing longer-lasting affect, the experiment could be repeated with the same individuals over multiple timepoints to check the stability of both questionnaire and judgement bias task responses.

In conclusion, we confirmed that self-reported negative affect is associated with negatively biased decision-making under ambiguity in humans, supporting the generality of this finding. We also found that this negative bias appears to arise during the decision-making process as opposed to being a result of an *a priori* bias towards making the ‘pessimistic’ response. However, contrary to our hypothesis, our results did not provide sufficient evidence for an association between self-reported dispositional pessimism and ‘pessimistic’ responses in the judgement bias task. Due to a strong significant correlation between self-reported anxiety and depression symptoms it was not possible to definitively disentangle the respective effects of anxiety and depression on judgement bias task responses.

## Additional File

The additional file for this article can be found as follows:

10.5334/cpsy.167.s1 Supplementary Material. Details of trial-level analysis, drift-diffusion model validation, and supplementary figures.

## Data Availability

All code and data to reproduce the analyses in this paper can be obtained at: https://doi.org/10.17605/OSF.IO/5JH6G.
