# Supplementary material for: The Effects of Negative Affect and Dispositional Pessimism on Decision-Making Under Ambiguity
Source: Comput Psychiatr. 2026 Oct 27;10(1):219–36. doi: 10.5334/cpsy.167 (PMC13722012; doi:10.5334/cpsy.167)
Supplement: Supplementary file 1 [file cpsy-10-1-167-s1.pdf]

Supplementary material

Trial-level analysis

To further investigate whether self-reported anxiety, depression, and pessimism, were significantly associated with responses in the judgement bias task, trial-level analyses were conducted using generalised linear mixed models (GLMMs). The GLMMs were run using the lme4 package in R, with a binomial error structure and logit link function. In all models the dependent variable was response (coded 0/1 where 0 represents the ‘pessimistic’ response and 1 represents the ‘optimistic’ response). Stimulus type, block, trial number, age, and sex were included as fixed factors in all models. Due to strong correlations between questionnaire scores, separate models were fit for each questionnaire measure (GAD-7, PHQ-8, and LOT-RP). Participant ID was also included as a random factor in all models. Standardised z-scores were used for all continuous predictors to improve model fitting and models were fitted using the bobyqa optimiser to ensure convergence. Likelihood ratio tests were used to assess the significance of fixed effects. Model fit was compared using Akaike Information Criterion (AIC). To investigate the effects of sex and stimulus on responses in the judgement bias task, post-hoc comparisons were conducted using a simultaneous pairwise Tukey procedure for general linear hypotheses with the R package ‘multcomp’ (Hothorn et al., 2008). Data and code are available here: https://doi.org/10.17605/OSF.IO/5JH6G

There was no evidence of a significant effect of pessimism (LOT-RP) score on responses (LRT = 1, $p = 0.232$), however both anxiety ($\beta \pm SE = -0.064 \pm 0.024$, LRT = 7, $p = 0.008$) and depression ($\beta \pm SE = -0.060 \pm 0.024$, LRT = 6, $p = 0.013$) scores were significantly negatively associated with the number of ‘optimistic’ responses made in the judgement bias task. These findings are consistent with the main analysis findings, where we showed that individuals who reported higher anxiety and depression scores made a significantly lower proportion of ‘optimistic’ responses to ambiguous stimuli.

All further results are reported using the model containing the GAD-7 questionnaire, as this model had the lowest AIC (GAD-7 AIC: 101,819.0; PHQ-8 AIC: 101,819.9; LOT-RP AIC: 101,824.6). The model revealed significant effects of age, sex, stimulus type, block, and trial number on task responses. Supporting our main analysis findings, age and sex were both significantly related to task responses. The number of ‘optimistic’ responses made significantly decreased with age ($\beta \pm SE = -0.096 \pm 0.024$, LRT = 16, $p < 0.001$) and sex was a significant predictor of responses (LRT = 33, $p < 0.001$), with males making significantly more ‘optimistic’ responses than females ($z = 5.716$, $p < 0.001$). Stimulus type showed a strong significant effect on responses made (LRT = 63,868, $p < 0.001$), with more ‘optimistic’ responses made for the more positive stimuli. Specifically, compared to the Ambiguous Negative stimulus, the Negative stimulus was associated with significantly reduced ‘optimistic’ responding ($z = -119.423$, $p < 0.001$), whereas the Positive stimulus was associated with significantly increased ‘optimistic’ responding ($z = 90.599$, $p < 0.001$). There was no evidence of a significant difference in ‘optimistic’ responses between the Ambiguous Negative and Ambiguous Positive stimuli ($z = 2.496$, $p = 0.055$). There was also a significant effect of block ($\beta \pm SE = 0.234 \pm 0.031$, LRT = 56, $p < 0.001$), with more ‘optimistic’ responses made in the second block, and a significant effect of trial number ($\beta \pm SE = 0.197 \pm 0.016$, LRT = 150, $p < 0.001$) with more ‘optimistic’ responses made as trials went on.

Drift-diffusion model validation

Drift-diffusion modelling was conducted using the Python toolbox HDDM (version 1.0.1 with Python 3.9.13, Wiecki et al., 2013). All code and plots can be found here: https://doi.org/10.17605/OSF.IO/5JH6G

Parameter recovery

Parameter recovery analyses were conducted to assess whether the hierarchical drift diffusion model (HDDM) used in the main analyses could reliably recover participant-level parameters under the trial counts and model specification employed in the study. True participant-level parameters for drift rate (*v*), threshold separation (*a*), start point bias (*z*), and non-decision time (*t*) were used to simulate decision and reaction time data. Using these true parameters, simulated choice and reaction time datasets were generated. For each participant a dataset containing 120 trials was generated.

The simulated datasets were fitted with the same HDDM used for the empirical data. The model estimated drift rate (*v*), threshold separation (*a*), start point bias (*z*), and non-decision time (*t*), with participant-level parameters hierarchically constrained by group-level distributions. Model fitting was conducted in HDDM using Markov chain Monte Carlo sampling with 10,000 posterior samples and 500 burn-in samples.

At the participant level, recovery was generally strong. Pearson correlations between true and recovered parameters across participants were high for drift rate ($r = 0.938$), boundary separation ($r = 0.956$), and non-decision time ($r = 0.997$), indicating excellent recovery of individual differences for these parameters. Recovery of starting point (*z*) was weaker ($r = 0.627$), though still indicative of moderate correspondence between true and recovered values. Bias at the participant level was calculated as the difference between recovered and true values for each participant. Standardised bias (mean bias divided by the standard deviation of recovered estimates) was used to assess the practical magnitude of bias relative to inter-individual variability. Participant-level bias was small across all parameters. Mean bias (± SD) was 0.009 ± 0.177 for *v*, −0.022 ± 0.052 for *a*, 0.002 ± 0.009 for *t*, and −0.001 ± 0.021 for *z*. When expressed relative to the variability of recovered estimates, standardised bias was very small for all parameters (*v*: 0.02 SD; *a*: −0.13 SD; *t*: 0.02 SD; *z*: −0.03 SD).

Posterior predictive checks also confirmed that the fitted model reproduced key features of the simulated reaction-time distributions, supporting the adequacy of the model.

Hold-out validation

A hold-out validation procedure was used to assess whether DDM parameters estimated from half of each participant’s data generalised to responses made in the held-out trials. For each participant, data from ambiguous trials were randomly split into two equal halves. One half of the trials, the training set, was used to fit the DDM, while the remaining trials were withheld for validation. Model fitting was conducted in HDDM using Markov chain Monte Carlo sampling with 10,000 posterior samples and 500 burn-in samples. Participant-level parameter estimates for the four parameters (drift rate (*v*), threshold separation (*a*), start point bias (*z*), and non-decision time (*t*)) were extracted. These parameters were then used to generate simulated data. For each participant, the number of simulated trials matched the number of trials in the corresponding held-out test dataset to ensure comparability.

Model predictions were evaluated by comparing simulated data to observed behaviour in the held-out trials. Choice data was summarised as the proportion of ‘optimistic’ responses for each participant, and reaction time data was summarised as the median reaction time for

each participant. Predictive accuracy was quantified using Pearson correlation coefficients between the observed and predicted values for each participant.

Predicted choice behaviour was strongly correlated with observed choice proportions across participants (r = 0.747), indicating that the model captured individual differences in responses. Predictive accuracy was also high for reaction times, with a very strong correlation between predicted and observed median reaction times (r = 0.931), suggesting that the model also accurately captured individual differences in reaction times. Together, these results indicate that DDM parameters estimated from a subset of the data generalise well to unseen trials.

Supplementary figures

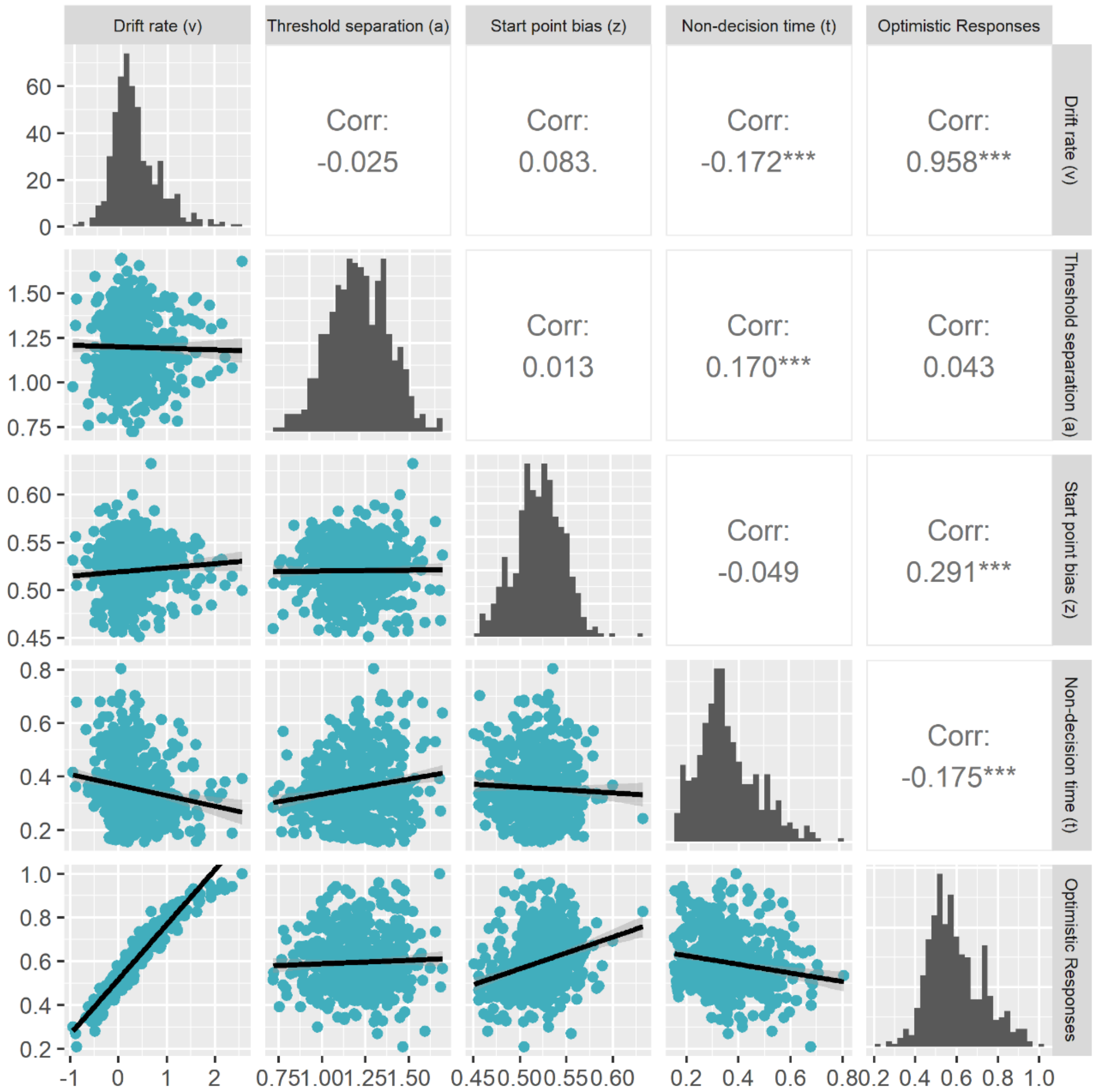

Figure S1: Associations between drift-diffusion model parameters and Optimistic Responses. *Diagonally from top left to bottom right: frequency distributions for each variable. Lower left: scatterplot with linear model lines ± shaded 95% confidence intervals. Upper right: Pearson correlation coefficient (***p < 0.001, .p < 0.10). n=516.*

Figure S1. alt text: Correlation matrix for drift-diffusion model parameters and optimistic responses.

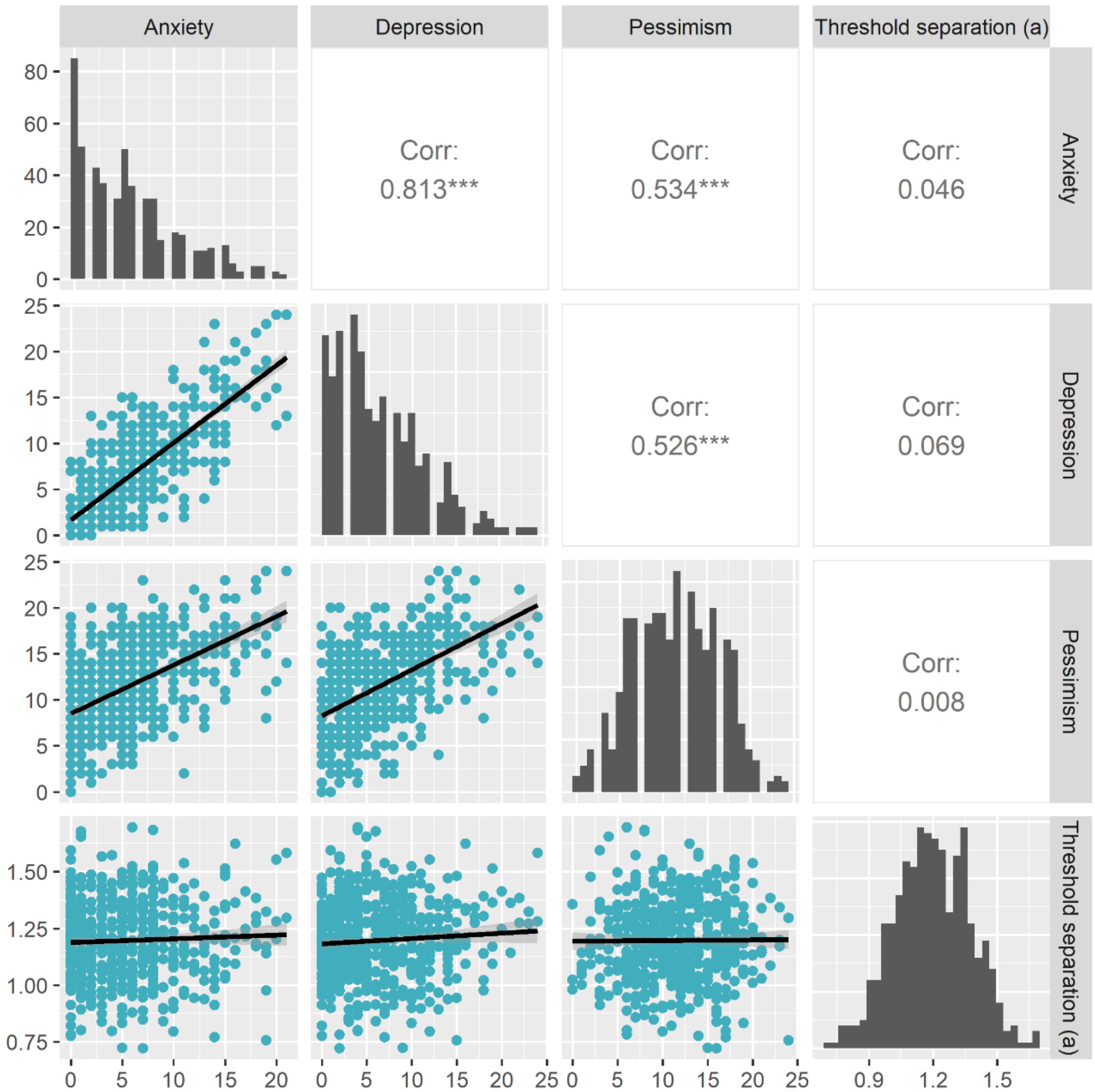

Figure S2: Associations between Anxiety, Depression, Pessimism, and Threshold separation (a). *Diagonally from top left to bottom right: frequency distributions for each variable. Lower left portion: scatterplots for each pair of variables with linear model lines ± shaded 95% confidence intervals. Upper right portion: Pearson correlation coefficients for each pair of variables (***p < 0.001). n=516.*

Figure S2. alt text: Correlation matrix for anxiety, depression, pessimism and threshold separation.

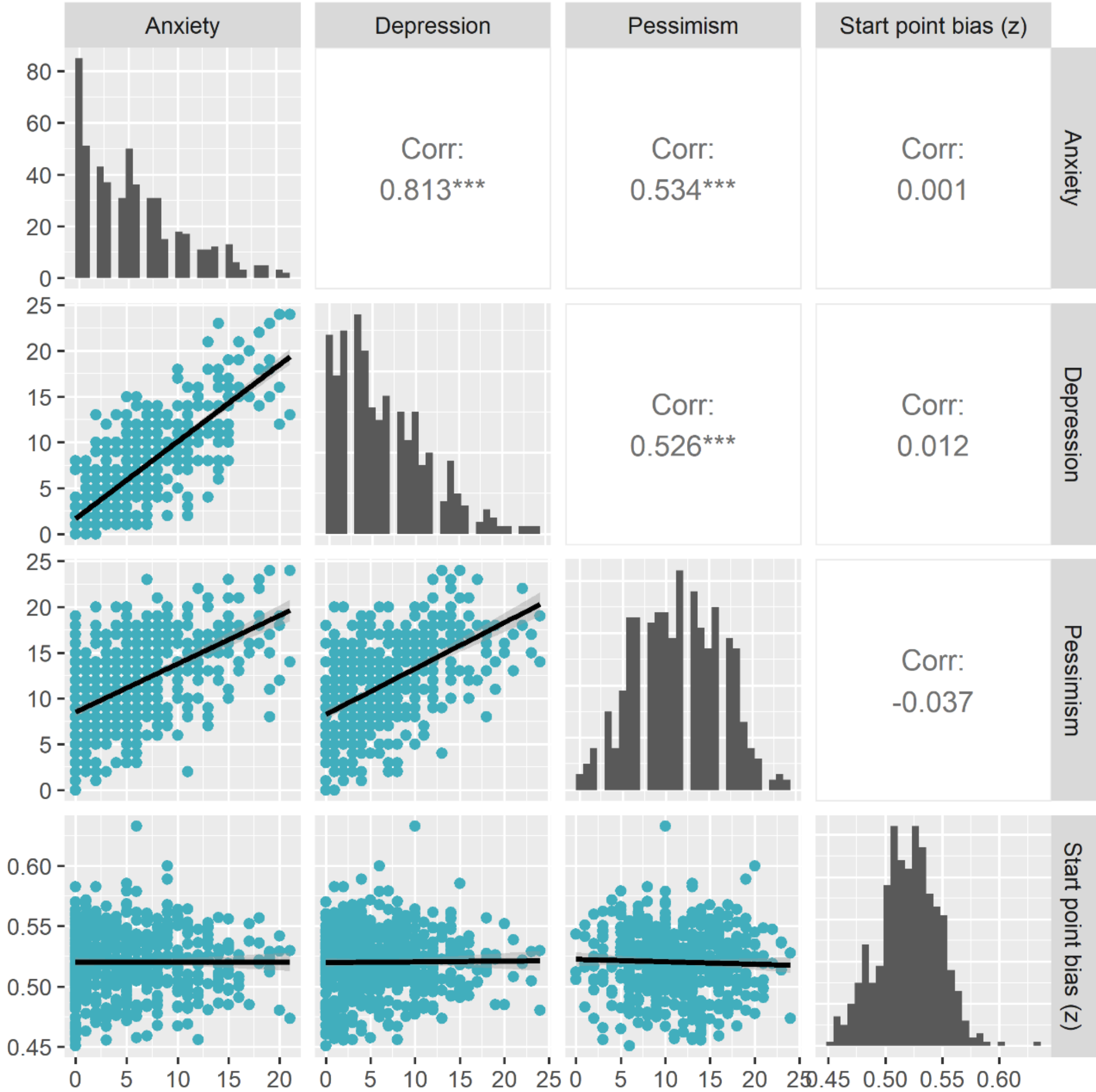

Figure S3: Associations between Anxiety, Depression, Pessimism, and Start point bias (z). *Diagonally from top left to bottom right: frequency distributions for each variable. Lower left portion: scatterplots for each pair of variables with linear model lines ± shaded 95% confidence intervals. Upper right portion: Pearson correlation coefficients for each pair of variables (***p < 0.001). n=516.*

Figure S3. alt text: Correlation matrix for anxiety, depression, pessimism and start point bias.

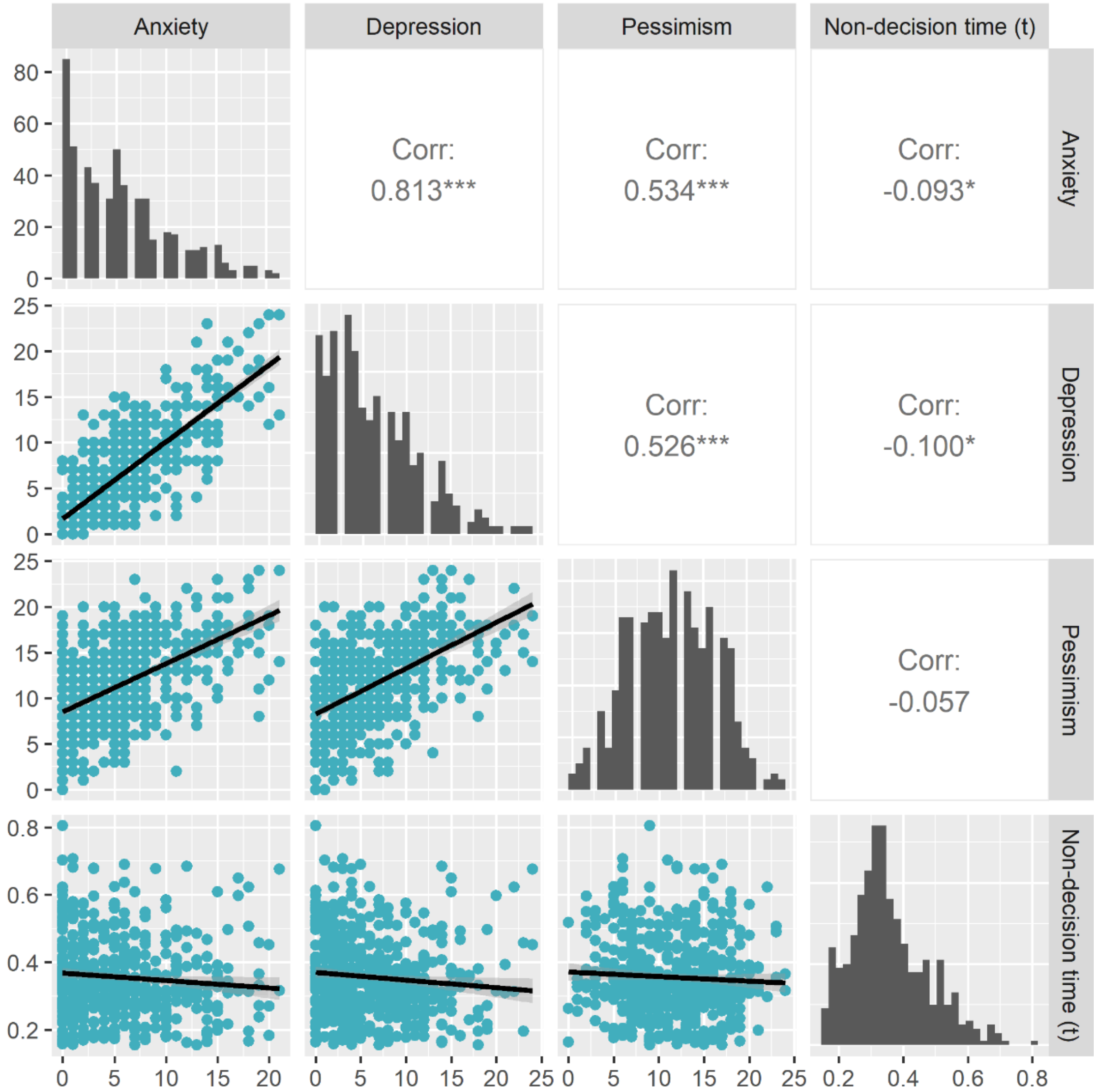

Figure S4: Associations between Anxiety, Depression, Pessimism, and Non-decision time (t). *Diagonally from top left to bottom right: frequency distributions for each variable. Lower left portion: scatterplots for each pair of variables with linear model lines ± shaded 95% confidence intervals. Upper right portion: Pearson correlation coefficients for each pair of variables (***p < 0.001, *p < 0.05). n=516.*

Figure S4. alt text: Correlation matrix for anxiety, depression, pessimism and non-decision time.
