# Supplementary material for: The Effects of Negative Affect and Dispositional Pessimism on Decision-Making Under Ambiguity
Source: Comput Psychiatr. 2026 Oct 27;10(1):219–36. doi: 10.5334/cpsy.167 (PMC13722012; doi:10.5334/cpsy.167)
Supplement: Peer review history

# Reviewer report: Round 1, Reviewer A

**DOI:** 10.5334/cpsy.167.r1

## submission-comments

This study investigates how self-reported negative affect (anxiety, depression) and trait level pessimism influence decision-making under ambiguity using a judgement bias task (online). The authors replicate previously reported findings that higher anxiety and depression scores are associated with fewer optimistic responses to ambiguous stimuli, and use modelling (the DDM) to show that this bias is driven by drift rate rather than start point bias. They also report a null finding for trait pessimism. The study is well-motivated and clearly written. The judgement bias task paradigm is well-established and the translational framing (from animal to human work) is a strength. However, there are a number of methodological and analytical concerns, detailed below.

Major Concerns

1. The near-perfect correlation between responses and drift rate (r = .96) reflects a circularity (double-dipping) problem that significantly undermines the DDM contribution of the paper. The drift rate parameter is estimated from the very same data that are then aggregated to compute the proportion of optimistic responses. Because drift rate in the DDM directly governs the probability of reaching one decision boundary versus the other, it is near-mechanically linked to choice proportions by construction. A higher drift rate toward the optimistic boundary will, by definition, produce a higher proportion of optimistic choices. Correlating drift rate with choice proportion from the same data is close to correlating a variable with itself. The r = .96 is therefore expected and not informative. This circularity matters because one central claim, that the negative bias arises “during the decision-making process” (drift rate) rather than “a priori” (starting bias), is built on showing that anxiety predicts drift rate. But if drift rate is essentially recapitulating the choice data, this claim is circular: it amounts to saying that people who chose pessimistically more often had a drift rate reflecting more pessimistic choices. The more meaningful question is whether the DDM decomposition reveals something that the raw choices do not.

To address this, the authors could use cross-validation/hold-out, e.g. fitting the DDM to one half of each participant’s trials (e.g. even vs. odd) and then test whether the estimated DDM parameters predict choice behaviour in the withheld trials. This would break the circularity by ensuring that the DDM parameters and the outcome variable are estimated from independent data. Without such a procedure, the DDM analyses do not provide evidence beyond what is already shown by the simple choice proportion analyses, and the mechanistic interpretation is not well-supported.

It’s possible I am confused; were the DDM parameters estimated from ambiguous trials only, or from all trials including unambiguous ones? This should be clearly stated. If unambiguous trials were included, the drift rate for those trials would be expected to be large (participants know the correct answer), and the averaging across trial types could complicate interpretation.

Additionally, I’d want to see correlations between *all* DDM parameters and responses, not just drift rate.

2. The multicollinearity issue between anxiety and depression (r = .81). The authors’ approach of running separate GLMs for each questionnaire score is understandable given the high correlation, but drawing conclusions about whether anxiety or depression is the better predictor based on marginal AIC differences (anxiety AIC: -654.675 vs. depression AIC: -654.178 -- a difference of ~0.5) that, to me, are so small they’re not meaningfully interpretable. The authors should either not distinguish between anxiety and depression as predictors and instead discuss them as a single construct, use a dimensionality reduction technique (e.g. PCA/factor analysis) to extract a common negative affect factor and test that, or orthogonalizing the variables, etc.. Making claims about anxiety being a marginally better predictor based on an AIC difference of less than 1 isn’t what I’d call sound.

3. The degree to which these parameters can be independently recovered given the task design and number of trials is not demonstrated. Given that the null result on z is a key finding (i.e., that negative affect does not influence a priori bias), showing that the model can actually detect a start point bias if one existed is key. Without parameter recovery, the null finding on z is uninterpretable (i.e., it could be a true null or it could reflect insufficient power/identifiability). I would strongly recommend a parameter recovery exercise to determine this.

4. This reviewer thinks that a trial-level mixed-effects logistic regression (with participant as a random effect) would be a more powerful and appropriate approach for binary choice data, as it would account for within-participant variability and would avoid the information loss inherent in collapsing 240 trials into a single proportion. This approach could also naturally incorporate trial-level predictors (e.g., stimulus type, block, trial number) and their interactions with the questionnaire measures.

Moderate Concerns

5. Due to the fixed task order, task performance could have influenced subsequent questionnaire responses. For example, if a participant performed poorly, could this have induced a temporarily more negative mood that inflated their anxiety or depression self-reports? While they ask about the past two weeks, momentary affect can influence self-report.

6. The correlations between anxiety/depression and optimistic responses explain only about 1% of the variance and regression coefficients are similarly small. How practically meaningful is a relationship that explains ~1% of variance? If the relationship between affect and judgement bias is this weak in humans who can directly report their affective state, what does this imply for the animal literature where affect is inferred? I think this deserves a more space.

7. The threshold separation finding (depression predicting increased threshold) should be interpreted cautiously because the p-value is .045, which is marginal, and this was the best model selected from four competing models via AIC (note my point above on the AICs). Given the number of GLMs run across the four DDM parameters (16 models total), there is a multiple comparisons concern. Was this finding pre-registered? If not, it should be labelled as exploratory. Also, the AIC difference between the depression model and the null model is only 2, which is conventionally considered weak evidence (at best) for model selection.

8. Related to point 7, are all analyses pre-registered, and are all pre-registered analyses reported? It may be an error/mistake on my part but I could not find where the preregistration could be accessed; this should be included in any revision.

9. Data quality controls for the online experiment could be more thorough. Only 4 participants were excluded for poor performance (responding incorrectly on ≥50% of unambiguous trials). This is a very lenient exclusion criterion. Were there participants who, for instance, showed very fast response times across many trials suggesting button-mashing, or who showed no variability in their responses? Given that this was an online study with relatively low compensation (£1.80 + 20p), it would be helpful to report any additional data checks (e.g., attention checks). How do we know participants were genuinely engaging with the stimuli given the very brief 250ms presentation time in an uncontrolled online environment?

Minor Concerns / Exposition

10. The two ambiguous stimuli are treated as equivalent and collapsed together. However, were these compared statistically? If they differ, collapsing them may obscure meaningful variation. It would also be helpful to report the accuracy on unambiguous trials in the results (not just as an exclusion criterion) so the reader can verify that participants were performing the task correctly.

11. The sex difference in optimistic responses is interesting. What might explain this discrepancy with prior literature? Is it related to the task variant, the sample, or the online vs. lab setting? Could this be driven by differences in anxiety/depression prevalence across sexes? The authors control for sex in their models, but an exploratory mediation analysis could be informative.

12. The Discussion raises the interesting point about whether judgement bias responses should be called optimistic/pessimistic given the null LOT-R finding. I appreciated this but it’s undermined by the fact that the paper itself uses these terms throughout. If the authors truly believe the terminology is problematic, it might be worth adopting alternative terms (e.g. positively/negatively bias) throughout?

13. The paper uses HDDM (hierarchical DDM). It would be useful to report model convergence diagnostics. Were any checks done to verify that the MCMC chains converged? How many samples/burn-in were used?

## Recommendation

Revisions Required

# Reviewer report: Round 1, Reviewer B

**DOI:** 10.5334/cpsy.167.r2

## submission-comments

This paper tests the assumption that the affective bias commonly seen in disorders like depression is related to pessimism. They carried out an online study where participants completed the judgment bias task and were assessed for anxiety (GAD7) and depression (PHQ9) as well as dispositional pessimism. The analyzed overall bias on the task and fit decisions to a drift diffusion model. They did not find any relationship with pessimism either with overall bias or with any parameters of the computational model the model. They did, however, show that anxiety and depression are related to lower drift rate and higher threshold. Overall, the paper is well written, and the topic is interesting. There are some areas of the analysis that are unclear and need to be clarified or improved as well as some conceptual questions.

Major comments:

The task used here seems very similar to the probabilistic reward task developed by Pizzagalli. It’s unclear why the literature on this task, which includes several papers using drift diffusion modeling, has not been incorporated into the introduction or discussion.

The conclusion mostly restates the results. More interpretation and more connections to related literature would strengthen the paper.

GAD7 and PHQ9 are highly correlated; therefore, it doesn’t make sense to first test a combined analysis where these are both in the regression. It’s unclear what this combined GLM is testing since any shared variance will not be included in the effects of the questionnaires.

Relatedly, the GAD7 and PHQ9 do not measure separate constructs. If the authors want to measure anxiety versus depression, it would be better to use questionnaires developed for this, such as those based on the tripartite model of anxiety and depression.

In table 1 it’s unclear where the AIC values are coming from. Are these from the regression or from the DDM? It’s also unclear why these numbers vary so widely with different drift diffusion model parameters.

It appears that DDM parameters are first extracted from the model for each person and then analyzed in a regression. The software used here should have the ability to analyze the effects of questionnaires in the model itself, rather than having to carry out a two-stage analysis that decreases power.

Model checking for the drift diffusion model is needed – such as posterior predictive checks and parameter recovery.

Minor:

Please state the correlation between the GAD7 and PHQ9 in the results or methods rather than in the discussion.

The discussion states that there is no evidence for a relationship between pessimism and performance on this task; however, this is not correct. Instead, the authors could not reject the null hypothesis that there is no relationship. The authors could use Bayesian analyses if they wanted to be able to show that there was no relationship.

## Recommendation

Resubmit for Review

# Reviewer report: Round 2, Reviewer A

**DOI:** 10.5334/cpsy.167.r3

## Recommendation:

Accept (No Revisions Necessary)

## Comments/Explanation (required). Please include references to any minor or major revisions required, as well as addressing any issues with language clarity. Authors will see these comments:

I thank the authors for their responsive edits. I do not have any further comments.

## Consent to Publish

Consent Given

# Reviewer report: Round 2, Reviewer B

**DOI:** 10.5334/cpsy.167.r4

## Recommendation:

Accept with Minor Revision

## Comments/Explanation (required). Please include references to any minor or major revisions required, as well as addressing any issues with language clarity. Authors will see these comments:

The authors have thoughtfully addressed my most serious concerns, which strengthen confidence in the results. A minor revision would allow authors to address outstanding issues such as language surrounding the multicollinearity between anxiety and depression measures and other minor issues, all detailed in my PDF review.

## Consent to Publish

Consent Given

# Reviewer report: Round 1, Author Response

**DOI:** 10.5334/cpsy.167.r5

Dear Dr Dongil Chung,

Ref: The Effects of Negative Affect and Dispositional Pessimism on Decision-Making Under Ambiguity

We thank you and the reviewers for your comments on the manuscript and detail below how we have addressed each of these. We hope that our work is now acceptable for publication.

Yours sincerely,

Molly Davidson, Vikki Neville, John Fennell, Elizabeth Paul, Iain Gilchrist, Michael Mendl

## Reviewer A:

**This study investigates how self-reported negative affect (anxiety, depression) and trait level pessimism influence decision-making under ambiguity using a judgement bias task (online). The authors replicate previously reported findings that higher anxiety and depression scores are associated with fewer optimistic responses to ambiguous stimuli, and use modelling (the DDM) to show that this bias is driven by drift rate rather than start point bias. They also report a null finding for trait pessimism. The study is well-motivated and clearly written. The judgement bias task paradigm is well-established and the translational framing (from animal to human work) is a strength. However, there are a number of methodological and analytical concerns, detailed below**.

We thank this reviewer for their positive comments on the manuscript, as well as their helpful suggestions below.

**Major Concerns**

**1. The near-perfect correlation between responses and drift rate (r = .96) reflects a circularity (double-dipping) problem that significantly undermines the DDM contribution of the paper. The drift rate parameter is estimated from the very same data that are then aggregated to compute the proportion of optimistic responses. Because drift rate in the DDM directly governs the probability of reaching one decision boundary versus the other, it is near-mechanically linked to choice proportions by construction. A higher drift rate toward the optimistic boundary will, by definition, produce a higher proportion of optimistic choices. Correlating drift rate with choice proportion from the same data is close to correlating a variable with itself. The r = .96 is therefore expected and not informative. This circularity matters because one central claim, that the negative bias arises “during the decision-making process” (drift rate) rather than “a priori” (starting bias), is built on showing that anxiety predicts drift rate. But if drift rate is essentially recapitulating the choice data, this claim is circular: it amounts to saying that people who chose pessimistically more often had a drift rate reflecting more pessimistic choices. The more meaningful question is whether the DDM decomposition reveals something that the raw choices do not**.

## Author’s response

We agree that the strong correlation between the proportion of ‘optimistic’ responses to ambiguous stimuli in the judgement bias task (Optimistic Responses) and drift rate is expected, given that the drift rate parameter directly affects the likelihood of reaching one decision boundary over the other. However, the Optimistic Responses measure only accounts for the choice data, whereas the DDM allows the choice and associated reaction time data to be investigated simultaneously. Combining choice and reaction time data, through the use of the DDM, gives a fuller picture of the decisions being made, for example a fast ‘optimistic’ response reflects a different decision process to a slow ‘optimistic’ response, despite the ultimate choice data being the same. Here we are using the DDM to specifically investigate whether a negative bias is more likely to occur due to a pre-existing (start point) bias to select the ‘pessimistic’ response prior to stimulus presentation or to arise during the decision-making process (represented by a negative drift rate).

Consider data which show a negative judgement bias i.e. there is a higher proportion of ‘pessimistic’ responses than ‘optimistic’ responses. If this was generated by an *a priori* bias, it would be reflected in a DDM by a relative start point parameter value of under 0.5 (with start point = 0.5 reflecting no *a priori* bias) due to the participant making the ‘pessimistic’ response more quickly and more often. Conversely, if the participant had similar reaction time profiles for both responses but responded ‘pessimistically’ more often, this would be reflected by a negative drift rate (with drift rate = 0 reflecting no drift towards either boundary) and suggest that the negative bias was generated during the decision-making process.

Whilst we observed a strong correlation between drift rate and Optimistic Responses, we suggest that the drift-diffusion modelling extends our findings by capturing choice and reaction time data simultaneously and therefore provides support for our claim that the negative bias associated with higher self-reported negative affect is more likely to arise during the decision-making process (related to the drift rate) rather than *a priori* (related to the start point bias).

We have updated our phrasing to be more cautious with our interpretation:

Lines 21-24: “Drift-diffusion modelling revealed that the more negative (less ‘optimistic’) judgement bias associated with self-reported anxiety and depression, could be explained by a bias generated during the decision-making process, represented by a lower drift rate.”

Lines 91-94: “One question which drift-diffusion modelling can be used to address is whether a bias towards one response is more likely to occur due to a pre-existing (start point) bias towards one option prior to stimulus presentation, or due to a difference in the rate of evidence accumulation (drift rate) during the decision-making process.”

## Reviewer A:

**To address this, the authors could use cross-validation/hold-out, e.g. fitting the DDM to one half of each participant’s trials (e.g. even vs. odd) and then test whether the estimated DDM parameters predict choice behaviour in the withheld trials. This would break the circularity by ensuring that the DDM parameters and the outcome variable are estimated from independent data. Without such a procedure, the DDM analyses do not provide evidence beyond what is already shown by the simple choice proportion analyses, and the mechanistic interpretation is not well-supported**.

## Author’s response

We have now conducted a hold-out validation exercise at the participant level as suggested and confirmed good predictive performance for held-out data. The code and associated plots for the hold-out validation analysis are available here: https://doi.org/10.17605/OSF.IO/5JH6G

This is referenced in the DDM methods section:

Lines 296-298: “[…] and a hold-out validation procedure confirmed good predictive performance for held-out data (see supplementary material for details).”

With full details available in the supplementary material:

“A hold-out validation procedure was used to assess whether DDM parameters estimated from half of each participant’s data generalised to responses made in the held-out trials. For each participant, data from ambiguous trials were randomly split into two equal halves. One half of the trials, the training set, was used to fit the DDM, while the remaining trials were withheld for validation. Model fitting was conducted in HDDM using Markov chain Monte Carlo sampling with 10,000 posterior samples and 500 burn in samples. Participant-level parameter estimates for the four parameters (drift rate (*v*), threshold separation (*a*), start point bias (*z*), and non-decision time (*t*)) were extracted. These parameters were then used to generate simulated data. For each participant, the number of simulated trials matched the number of trials in the corresponding held-out test dataset to ensure comparability.

Model predictions were evaluated by comparing simulated data to observed behaviour in the held-out trials. Choice data was summarised as the proportion of ‘optimistic’ responses for each participant, and reaction time data was summarised as the median reaction time for each participant. Predictive accuracy was quantified using Pearson correlation coefficients between the observed and predicted values for each participant.

Predicted choice behaviour was strongly correlated with observed choice proportions across participants (r = 0.747), indicating that the model captured individual differences in responses. Predictive accuracy was also high for reaction times, with a very strong correlation between predicted and observed median reaction times (r = 0.931), suggesting that the model also accurately captured individual differences in reaction times. Together, these results indicate that DDM parameters estimated from a subset of the data generalise well to unseen trials.”

## Reviewer A:

**It’s possible I am confused; were the DDM parameters estimated from ambiguous trials only, or from all trials including unambiguous ones? This should be clearly stated. If unambiguous trials were included, the drift rate for those trials would be expected to be large (participants know the correct answer), and the averaging across trial types could complicate interpretation**.

## Author’s response

Yes, the DDM parameters were estimated from ambiguous trials only. We apologise that this was unclear and have added in a sentence to state this:

Lines 286-287: “Only data for the ambiguous judgement bias task trials were included in the model.”

## Reviewer A:

Additionally, I’d want to see correlations between *all* DDM parameters and responses, not just drift rate.

## Author’s response

Figure 6 has been moved to the supplementary material (now Figure S1) and updated to show correlations between all the DDM parameters (*v, a, z, t*) and Optimistic Responses, and the figure legend and alt text have been changed:

Supplementary material: “*Figure S1: Associations between drift-diffusion model parameters and Optimistic Responses. Diagonally from top left to bottom right: frequency distributions for each variable. Lower left: scatterplot with linear model lines* ± *shaded 95% confidence intervals. Upper right: Pearson correlation coefficient* (***p < 0.001, .p < 0.10). *n=516*.

*Figure S1. alt text: Correlation matrix for drift-diffusion model parameters and optimistic responses.”*

Additional figures have also been added to the supplementary material to show the correlations between the other DDM parameters (*a, z, t*) and the questionnaire scores.

## Reviewer A:

**2. The multicollinearity issue between anxiety and depression (r = .81). The authors’ approach of running separate GLMs for each questionnaire score is understandable given the high correlation, but drawing conclusions about whether anxiety or depression is the better predictor based on marginal AIC differences (anxiety AIC: -654.675 vs. depression AIC: -654.178 -- a difference of ~0.5) that, to me, are so small they’re not meaningfully interpretable. The authors should either not distinguish between anxiety and depression as predictors and instead discuss them as a single construct, use a dimensionality reduction technique (e.g. PCA/factor analysis) to extract a common negative affect factor and test that, or orthogonalizing the variables, etc.. Making claims about anxiety being a marginally better predictor based on an AIC difference of less than 1 isn’t what I’d call sound**.

## Author’s response

Anxiety and depression were analysed separately rather than combined using dimensional reduction, as our aim was to treat them as distinct constructs. We anticipated a high correlation between GAD-7 and PHQ-8 scores and preregistered that we would run separate GLMs for each questionnaire score. This approach was followed in the current analyses.

We agree that the difference in model fit between the anxiety and depression GLMs, as indicated by AIC values, is too small to be meaningfully interpreted. We have now stated the AIC values in the judgement bias results section, rather than the discussion, and referred to them being similar:

Lines 338-340: “Model fit was similar for the anxiety and depression models (AICs = –654.675 and –654.178, respectively).”

We have also updated the relevant paragraph in the discussion to reflect this:

Lines 500-510: “A secondary aim of this study was to explore whether anxiety or depression symptoms drive ‘pessimistic’ decision-making in the judgement bias task. We found anxiety and depression scores to be very highly correlated. This meant that they could not be included in the same model, and when considered separately, both anxiety and depression scores were significantly associated with a negative judgement bias. Due to negligible differences in model fit, it was not possible to distinguish between anxiety and depression here, so instead we suggest that ‘pessimistic’ decision-making is related to negative affect which is likely being captured by both questionnaires. When analysed separately, anxiety and depression scores were also both significantly associated with a lower drift rate. Again, we observed only a small difference in model fit, so conclude that the lower drift rate is associated with negative affect, rather than anxiety or depression specifically.”

## Reviewer A:

**3. The degree to which these parameters can be independently recovered given the task design and number of trials is not demonstrated. Given that the null result on z is a key finding (i.e., that negative affect does not influence a priori bias), showing that the model can actually detect a start point bias if one existed is key. Without parameter recovery, the null finding on z is uninterpretable (i.e., it could be a true null or it could reflect insufficient power/identifiability). I would strongly recommend a parameter recovery exercise to determine this**.

We appreciate the importance of parameter recovery for interpreting the model findings. HDDM is widely used for drift-diffusion modelling, and the hierarchical nature of the model has been shown to aid parameter recovery, particularly when trial numbers are low (Wiecki et al., 2013). To address this concern directly, we have now conducted a parameter recovery exercise. This demonstrated good recovery of parameters at the participant level, including the starting point parameter (*z*), indicating that the model is capable of detecting such effects given our task design. The code and associated plots for the parameter recovery analysis are available here: https://doi.org/10.17605/OSF.IO/5JH6G

This is referenced in the DDM methods section:

Lines 296-298: “Parameter recovery checks showed good recovery, and a hold-out validation procedure confirmed good predictive performance for held-out data (see supplementary material for details).”

With full details available in the supplementary material:

“Parameter recovery analyses were conducted to assess whether the hierarchical drift diffusion model (HDDM) used in the main analyses could reliably recover participant level parameters under the trial counts and model specification employed in the study. True participant level parameters for drift rate (*v*), threshold separation (*a*), start point bias (*z*), and non decision time (*t*) were used to simulate decision and reaction time data. Using these true parameters, simulated choice and reaction time datasets were generated. For each participant a dataset containing 120 trials was generated.

The simulated datasets were fitted with the same HDDM used for the empirical data. The model estimated drift rate (*v*), threshold separation (*a*), start point bias (*z*), and non decision time (*t*), with participant level parameters hierarchically constrained by group level distributions. Model fitting was conducted in HDDM using Markov chain Monte Carlo sampling with 10,000 posterior samples and 500 burn in samples.

At the participant level, recovery was generally strong. Pearson correlations between true and recovered parameters across participants were high for drift rate (r = 0.938), boundary separation (r = 0.956), and non decision time (r = 0.997), indicating excellent recovery of individual differences for these parameters. Recovery of starting point (*z*) was weaker (r = 0.627), though still indicative of moderate correspondence between true and recovered values. Bias at the participant level was calculated as the difference between recovered and true values for each participant. Standardised bias (mean bias divided by the standard deviation of recovered estimates) was used to assess the practical magnitude of bias relative to inter individual variability. Participant level bias was small across all parameters. Mean bias (± SD) was 0.009 ± 0.177 for *v*, –0.022 ± 0.052 for *a*, 0.002 ± 0.009 for *t*, and –0.001 ± 0.021 for *z*. When expressed relative to the variability of recovered estimates, standardised bias was very small for all parameters (*v*: 0.02 SD; *a*: –0.13 SD; *t*: 0.02 SD; *z*: –0.03 SD).

Posterior predictive checks also confirmed that the fitted model reproduced key features of the simulated reaction time distributions, supporting the adequacy of the model.”

## Reviewer A:

**4. This reviewer thinks that a trial-level mixed-effects logistic regression (with participant as a random effect) would be a more powerful and appropriate approach for binary choice data, as it would account for within-participant variability and would avoid the information loss inherent in collapsing 240 trials into a single proportion. This approach could also naturally incorporate trial-level predictors (e.g., stimulus type, block, trial number) and their interactions with the questionnaire measures**.

We agree that a trial-level mixed effects logistic regression is a powerful approach for analysing this data. Our original analysis approach focused on the proportion of ‘optimistic’ responses to ambiguous stimuli as a concise and interpretable summary measure which we aimed to relate to questionnaire measures at the participant level. This approach was chosen to maintain consistency with the literature, allowing for comparisons with other human and animal studies using similar tasks. We have maintained the original approach in the manuscript following our preregistered plan, and in addition to address this concern, we have conducted an additional trial-level mixed effects logistic regression analysis. Due to strong correlations between the three questionnaire measures, we ran three separate GLMMs, each containing one of the questionnaire scores as a factor. The main findings were consistent with those from the original analyses; there was no evidence of a significant effect of pessimism score on responses, however both anxiety and depression scores were significantly negatively associated with the number of ‘optimistic’ responses made in the judgement bias task. The code and associated plots for the trial-level analysis are available here: https://doi.org/10.17605/OSF.IO/5JH6G

This is referenced in the statistical analyses methods section:

Lines 278-282: “The Optimistic Responses summary score was used to make our analysis comparable to ‘standard’ analyses of human and animal judgement bias tasks, however recognising the loss of information with this approach, additional trial-level analyses were performed to check whether similar results were observed from the complete dataset (see supplementary material).”

And the judgement bias results section:

Lines 340-344: “Findings from the trial-level analyses were consistent with those from the main analyses; there was no evidence of a significant effect of pessimism score on task responses, however both anxiety and depression scores were significantly negatively associated with the number of ‘optimistic’ responses made in the judgement bias task (see supplementary material).”

With full details available in the supplementary material:

“To further investigate whether self-reported anxiety, depression, and pessimism, were significantly associated with responses in the judgement bias task, trial-level analyses were conducted using generalised linear mixed models (GLMMs). The GLMMs were run using the lme4 package in R, with a binomial error structure and logit link function. In all models the dependent variable was response (coded 0/1 where 0 represents the ‘pessimistic’ response and 1 represents the ‘optimistic’ response). Stimulus type, block, trial number, age, and sex were included as fixed factors in all models. Due to strong correlations between questionnaire scores, separate models were fit for each questionnaire measure (GAD-7, PHQ-8, and LOT-RP). Participant ID was also included as a random factor in all models. Standardised z-scores were used for all continuous predictors to improve model fitting and models were fitted using the bobyqa optimiser to ensure convergence. Likelihood ratio tests were used to assess the significance of fixed effects. Model fit was compared using Akaike Information Criterion (AIC). To investigate the effects of sex and stimulus on responses in the judgement bias task, post-hoc comparisons were conducted using a simultaneous pairwise Tukey procedure for general linear hypotheses with the R package ‘multcomp’ (Hothorn et al., 2008). Data and code are available here: https://doi.org/10.17605/OSF.IO/5JH6G

There was no evidence of a significant effect of pessimism (LOT-RP) score on responses (LRT = 1, p = 0.232), however both anxiety (β ± SE = –0.064 ± 0.024, LRT = 7, p = 0.008) and depression (β ± SE = –0.060 ± 0.024, LRT = 6, p = 0.013) scores were significantly negatively associated with the number of ‘optimistic’ responses made in the judgement bias task. These findings are consistent with the main analysis findings, where we showed that individuals who reported higher anxiety and depression scores made a significantly lower proportion of ‘optimistic’ responses to ambiguous stimuli.

All further results are reported using the model containing the GAD-7 questionnaire, as this model had the lowest AIC (GAD-7 AIC: 101,819.0; PHQ-8 AIC: 101,819.9; LOT-RP AIC: 101,824.6). The model revealed significant effects of age, sex, stimulus type, block, and trial number on task responses. Supporting our main analysis findings, age and sex were both significantly related to task responses. The number of ‘optimistic’ responses made significantly decreased with age (β ± SE = –0.096 ± 0.024, LRT = 16, p < 0.001) and sex was a significant predictor of responses (LRT = 33, p < 0.001), with males making significantly more ‘optimistic’ responses than females (z = 5.716, p < 0.001). Stimulus type showed a strong significant effect on responses made (LRT = 63,868, p < 0.001), with more ‘optimistic’ responses made for the more positive stimuli. Specifically, compared to the Ambiguous Negative stimulus, the Negative stimulus was associated with significantly reduced ‘optimistic’ responding (z = -119.423, p < 0.001), whereas the Positive stimulus was associated with significantly increased ‘optimistic’ responding (z = 90.599, p < 0.001). There was no evidence of a significant difference in ‘optimistic’ responses between the Ambiguous Negative and Ambiguous Positive stimuli (z = 2.496, p = 0.055). There was also a significant effect of block (β ± SE = 0.234 ± 0.031, LRT = 56, p < 0.001), with more ‘optimistic’ responses made in the second block, and a significant effect of trial number (β ± SE = 0.197 ± 0.016, LRT = 150, p < 0.001) with more ‘optimistic’ responses made as trials went on.”

## Reviewer A:

**Moderate Concerns**

**5. Due to the fixed task order, task performance could have influenced subsequent questionnaire responses. For example, if a participant performed poorly, could this have induced a temporarily more negative mood that inflated their anxiety or depression self-reports? While they ask about the past two weeks, momentary affect can influence self-report**.

## Author’s response

We agree that the fixed study order means that task performance could have influenced subsequent questionnaire responses. We had briefly mentioned the potential effects of affective states on questionnaire responses (lines 494-496), however we have now linked this to the fixed task order and have acknowledged this limitation explicitly. We also clarify our rationale for using a fixed task order and administering the judgement bias task first:

Lines 574-589: “While our study focused on the relationship between self-reported anxiety and depression during the past two weeks and judgement bias performance, responses may also have been influenced by shorter-term changes in participants’ affective states (Loewenstein & Lerner, 2003). Over the short test session, we expected that participants’ moods would be relatively stable and therefore would predictably influence task and questionnaire responses over the whole session. However, due to the fixed study order it is possible that task performance could have influenced subsequent questionnaire responses. For example, poorer performance on the judgement bias task may have induced a temporarily more negative mood, potentially inflating self-reported symptoms of anxiety or depression. A fixed study order was used to facilitate the investigation of individual differences, and the judgement bias task was completed first as we believed it likely that explicitly directing participants’ attention to their affective state during self-report could attenuate the extent to which affect influenced decision-making in the judgement bias task (Kassam & Mendes, 2013). Alternatively, completing questionnaires assessing anxiety, depression, and pessimism may induce a negative affective state which could influence task performance. Nonetheless, potential order effects should be considered when interpreting our findings.”

## Reviewer A:

**6. The correlations between anxiety/depression and optimistic responses explain only about 1% of the variance and regression coefficients are similarly small. How practically meaningful is a relationship that explains ~1% of variance? If the relationship between affect and judgement bias is this weak in humans who can directly report their affective state, what does this imply for the animal literature where affect is inferred? I think this deserves a more space**.

## Author’s response

We have added an additional paragraph to the discussion to explore this point further, particularly emphasising the translational implications of small effect sizes observed in human judgement bias task studies:

Lines 533-551: “One concern highlighted by the small effect sizes observed here and in other human judgement bias studies (e.g. Daniel-Watanabe et al., 2022; Armbruster-Genç et al., 2024), is how sensitive and selective these tasks are to detecting and measuring negative (or positive) affect. Currently it is unclear whether small or null effects in these studies reflect limited sensitivity or specificity of the judgement bias tasks themselves, limitations of the self-report measures used to capture affect, or both. These human back-translated judgement bias task studies are relatively new therefore further work is needed to disentangle these possibilities and evaluate their reliability and validity for translational research (Pike et al., 2021). If the relationship between affect and judgement bias is genuinely weak, even in humans who can explicitly report their affective state (albeit imperfectly), this necessarily limits how strongly judgement bias outcomes can be interpreted in animals, where affect must be inferred indirectly. In addition, animal studies are likely to be underpowered, particularly when weaker affect manipulations are used, as it is difficult to achieve large enough sample sizes to detect small effects. Importantly, however, meta-analyses of animal judgement bias studies indicate that both pharmacological and non-pharmacological affect manipulations produce small to small-to-moderate effects in the predicted direction (Lagisz et al., 2020; Neville et al., 2020). Taken together, these findings suggest that judgement bias is sensitive to affective manipulations, but potentially with limited precision, reinforcing the need to interpret it as one component within a broader framework of cognitive, behavioural, and physiological measures of affect.”

## Reviewer A:

**7. The threshold separation finding (depression predicting increased threshold) should be interpreted cautiously because the p-value is .045, which is marginal, and this was the best model selected from four competing models via AIC (note my point above on the AICs). Given the number of GLMs run across the four DDM parameters (16 models total), there is a multiple comparisons concern. Was this finding pre-registered? If not, it should be labelled as exploratory. Also, the AIC difference between the depression model and the null model is only 2, which is conventionally considered weak evidence (at best) for model selection**.

## Author’s response

We have updated the threshold separation results section to highlight that this finding is marginal:

Lines 407-408: “Threshold separation showed a marginal positive association with depression scores (β±SE = 0.003 ± 0.002, LRT = 4.020, p = 0.045, Figure S2).”

We have also updated the methods section to state that all analyses relating to drift-diffusion modelling were not preregistered and are exploratory:

Lines 313-314: “All analyses relating to drift-diffusion modelling were not preregistered and are purely exploratory.”

And emphasised this in the discussion:

Lines 459-461: “All drift-diffusion model analyses were not preregistered meaning any findings are exploratory and should be interpreted cautiously.”

## Reviewer A:

**8. Related to point 7, are all analyses pre-registered, and are all pre-registered analyses reported? It may be an error/mistake on my part but I could not find where the preregistration could be accessed; this should be included in any revision**.

## Author’s response

The preregistration can be found on the OSF here: https://osf.io/shvfr and the link is included in the methods section:

Line 152: “This experiment was preregistered on the OSF on the 28th of June 2021: https://osf.io/shvfr”

Not all of the analyses were preregistered, however all of the preregistered analyses are reported. We agree that this was unclear in the original manuscript and have made the following changes:

Lines 152-153: “All preregistered analyses are reported, and exploratory analyses are labelled as such.”

Lines 313-314: “All analyses relating to drift-diffusion modelling were not preregistered and are purely exploratory.”

## Reviewer A:

**9. Data quality controls for the online experiment could be more thorough. Only 4 participants were excluded for poor performance (responding incorrectly on ≥50% of unambiguous trials). This is a very lenient exclusion criterion. Were there participants who, for instance, showed very fast response times across many trials suggesting button-mashing, or who showed no variability in their responses? Given that this was an online study with relatively low compensation (£1.80 + 20p), it would be helpful to report any additional data checks (e.g., attention checks). How do we know participants were genuinely engaging with the stimuli given the very brief 250ms presentation time in an uncontrolled online environment?**

## Author’s response

To support participant engagement, the study was intentionally designed to be completed within a relatively short time frame (median study completion time: 16 minutes and 38 seconds). Participants received £1.80 for their time plus a 20p performance-related bonus, exceeding Prolific’s minimum hourly payment at the time of data collection. Participants were informed that the bonus depended on whether they responded correctly on one trial selected at random, and that an additional £10 bonus would be awarded to the highest-performing participant for every 100 completions; this bonus was paid as stated. In addition, to ensure that participants understood the stimuli and response contingencies, each testing block was preceded by a 20-trial training block consistently exclusively of unambiguous trials with immediate feedback. Participants who did not complete the whole study were assumed to have withdrawn and were excluded in line with our preregistered protocol (n=20).

Following our preregistration, trials with very short reaction times (<200ms), assumed to reflect accidental key presses (or “button mashing”), were excluded from the analysis (1321/145,600 or 0.91%). Similarly, data from trials where reaction times were too long (>2000ms), and therefore not likely to relate to the presented stimuli, were excluded (844/145,600 or 0.58%). In addition, four participants were excluded for poor task performance (responding incorrectly on 50% or more of either of the unambiguous stimuli trials). Although this exclusion criterion is relatively lenient, accuracy among the remaining participants was high: mean accuracy was 93% on negative unambiguous trials and 97% on positive unambiguous trials, well above chance levels, with expected variability across individuals (see Figure 2). All retained participants contributed sufficient ambiguous trial data for analysis (median = 120 trials, range = 59–120).

As a further quality control check, participants were asked at the end of the study “Did you experience any interruptions or technical difficulties whilst completing this study? If yes, please specify what happened”. No concerning responses were reported. While we acknowledge that explicit attention checks were not included, we note that the task design itself (relatively short completion time, payment and bonus incentives, forced-choice responses, training with feedback, etc.) promoted engagement. This is evidenced by expected reaction time distribution and high accuracy on the unambiguous trials, despite the task being completed online. To improve transparency of our data quality controls, the following text has been amended in the statistical analyses methods section:

Lines 254-264: “For the whole dataset, data from trials where the reaction times were very short (<200ms) were excluded from the analysis as these were assumed to be accidental key presses rather than deliberate responses (1321/145,600 or 0.907% excluded) (Myers et al., 2022). Similarly, data from trials where reaction times were too long (>2000ms), and therefore not likely to relate to the presented stimuli, were excluded from the analysis (844/145,600 or 0.580% excluded) (Myers et al., 2022 In addition, data from 4 participants were excluded due to poor performance in the task (responding incorrectly on 50% or more of either of the unambiguous stimuli trials). Mean accuracy for the remaining participants on negative unambiguous trials was 93% (1SD = 0.09) and on positive unambiguous trials was 97% (1SD = 0.05). The median number of ambiguous trials following the exclusion of trials with reaction times <200ms and >200ms was 120 (range: 59-120).”

## Reviewer A:

**Minor Concerns / Exposition**

**10. The two ambiguous stimuli are treated as equivalent and collapsed together. However, were these compared statistically? If they differ, collapsing them may obscure meaningful variation. It would also be helpful to report the accuracy on unambiguous trials in the results (not just as an exclusion criterion) so the reader can verify that participants were performing the task correctly**.

## Author’s response

We now report a direct comparison between responses to the two ambiguous stimuli. When comparing only response to the two ambiguous stimuli (Ambiguous Negative and Ambiguous Positive) using a paired-samples t-test, a small statistically significant difference (p = 0.032) was observed. Post-hoc comparisons of our trial-level analysis (see supplementary material), did not find a significant difference in ‘optimistic’ responses between the Ambiguous Negative and Ambiguous Positive stimuli (z = 2.496, p = 0.055). In addition, previous work has shown Gabor patches using these orientations cannot be characterised above chance levels (Iigaya et al., 2016). Based on this we have kept the combined ambiguous measure for subsequent analyses.

This information has been added into the methods:

Lines 228-239: “Responses to the two ambiguous stimuli (Ambiguous Negative and Ambiguous Positive) were compared using a paired-samples t-test, the assumptions of which were met; a Shapiro–Wilk test indicated that the difference scores did not deviate from normality (W = 0.998, p = 0.777) supported by visual inspection of the histogram and Q-Q plots. A statistically significant difference was observed (t(515) = –2.15, p = 0.032) albeit with a very small effect size (mean difference = –0.008; Cohen’s dz = –0.09, 95% CI [–0.18, –0.01]). No significant difference in ‘optimistic’ responses between the Ambiguous Negative and Ambiguous Positive stimuli was found in post-hoc comparisons of our trial-level analysis (z = 2.496, p = 0.055; see supplementary material). Based on this, and existing evidence that Gabor patch stimuli of these orientations cannot be reliably characterised above chance levels (Iigaya et al., 2016), the proportion of ‘optimistic’ responses were combined for both ambiguous stimuli to give each participant an ‘Optimistic Response’ score.”

We have also added information on the accuracy on unambiguous trials to the methods and results sections:

Methods:

Lines 261-263: “Mean accuracy for the remaining participants on negative unambiguous trials was 93% (1SD = 0.09) and on positive unambiguous trials was 97% (1SD = 0.05).”

Results:

Lines 318-324: “The mean proportion of ‘optimistic’ responses to the negative unambiguous stimulus was 0.07 (1SD = 0.09) and to the positive unambiguous stimuli was 0.97 (1SD = 0.05) (Figure 2). The mean proportions of ‘optimistic responses to the two ambiguous stimuli, Ambiguous Negative and Ambiguous Positive, were 0.59 (1SD = 0.14) and 0.60 (1SD = 0.14) respectively (Figure 2). For subsequent analyses, ambiguous responses were combined for each participant to give the proportion of ‘optimistic’ responses to ambiguous stimuli in the judgement bias task (Optimistic Responses); the mean Optimistic Responses value was 0.60 (1SD = 0.13). “

## Reviewer A:

**11. The sex difference in optimistic responses is interesting. What might explain this discrepancy with prior literature? Is it related to the task variant, the sample, or the online vs. lab setting? Could this be driven by differences in anxiety/depression prevalence across sexes? The authors control for sex in their models, but an exploratory mediation analysis could be informative**.

## Author’s response

We agree that the observed sex difference in the proportion of ‘optimistic’ responses made to ambiguous stimuli in the judgement bias task is interesting, but it was not a focus of the study and was not preregistered therefore it should be treated as an exploratory finding that warrants further investigation, especially as the existing literature on this topic is limited. We identified one similar study to which we could compare this finding; whilst we found that males made a significantly higher proportion of ‘optimistic’ responses to ambiguous stimuli than females, Daniel-Watanabe et al.’s (2022) study found no significant effect of sex. This discrepancy may relate to the type of task used (different stimuli, trial number, etc.), or sample demographics (different recruitment methods, geographical restrictions, etc.). Findings may also relate to what the tasks are actually measuring, for example our task may also be capturing risk-taking behaviour which has been shown to be higher in males than females (e.g. Byrnes et al., 1999; Charness & Gneezy, 2012). Evidence for sex effects on judgement bias in animal studies is also mixed; a meta-analysis of non-pharmacological judgement bias studies found that affect manipulations tended to have a larger effect in males than females (Lagisz et al., 2020), however the accompanying meta-analysis of pharmacological studies found that sex did not significantly modulate judgement bias (Neville et al., 2020). Whilst exploratory mediation analysis isn’t something we have applied here, it is a valuable suggestion for future studies which specifically want to look at the effects of sex on judgement bias.

We have added some additional sentences to the discussion to clarify that the observed judgement bias sex difference was an exploratory finding, and that it may relate to other non-affect related traits such as risk-taking behaviour:

Lines 560-565: “Whilst these age and sex effects are interesting, they were not a focus of the study and were not preregistered therefore they should be treated as exploratory findings that warrant further investigation, especially as the existing literature on this topic is limited. It is also likely that judgement bias tasks are capturing other non-affect related traits, such as risk-taking behaviour which has been shown to be higher in males than females (e.g. Byrnes et al., 1999; Charness & Gneezy, 2012).”

## Reviewer A:

**12. The Discussion raises the interesting point about whether judgement bias responses should be called optimistic/pessimistic given the null LOT-R finding. I appreciated this but it’s undermined by the fact that the paper itself uses these terms throughout. If the authors truly believe the terminology is problematic, it might be worth adopting alternative terms (e.g. positively/negatively bias) throughout?**

## Author’s response

We agree that the terminology should be carefully considered, particularly given the lack of evidence to reject the null hypothesis that self-reported pessimism is inversely associated with ‘optimistic’ responses in the judgement bias task. In the current paper, we have chosen to retain the terms ‘optimistic’ and ‘pessimistic’ throughout to maintain consistency with the existing human and animal judgement bias research literature, where these labels have been widely used to provide intuitive shorthand for task behaviour. We have now clarified in the introduction and discussion that these terms are used in an operational sense to describe task-specific response patterns rather than optimism and pessimism as broader traits:

Lines 79-82: “When referring to judgement bias task responses, we use the terms ‘optimistic’ and ‘pessimistic’ to maintain consistency with the literature, however they are used in an operational sense to describe task-specific response patterns rather than dispositional traits.”

Lines 452-454: “In the present paper, we continue to use the terms ‘optimistic’ and ‘pessimistic’ to remain consistent with the wider literature, however they are used operationally to describe task responses rather than broader personality traits.”

## Reviewer A:

**13. The paper uses HDDM (hierarchical DDM). It would be useful to report model convergence diagnostics. Were any checks done to verify that the MCMC chains converged? How many samples/burn-in were used?**

## Author’s response

We have now clarified the Markov chain Monte Carlo (MCMC) sampling details and convergence diagnostics used for the HDDM analyses in the drift-diffusion modelling methods section:

Lines 287-295:

“The model was fitted hierarchically using Markov chain Monte Carlo (MCMC) sampling and contained 4 parameters: drift rate (*v*), threshold separation (*a*), start point bias (*z*), and non-decision time (*t*). We ran 10,000 MCMC iterations, discarding the first 500 samples as burn-in. Starting values were optimised prior to sampling to facilitate convergence. Model convergence was assessed through visual inspection of trace plots and autocorrelation functions. These diagnostics indicated stable post burn in sampling, good mixing, and no evidence of systematic trends. In addition, posterior predictive checks indicated that the fitted model adequately reproduced the observed reaction time distributions, suggesting satisfactory convergence and model fit.”

Our HDDM code including the model trace plots is available here: https://doi.org/10.17605/OSF.IO/5JH6G

## Reviewer B:

**Reviewer 2:**

**This paper tests the assumption that the affective bias commonly seen in disorders like depression is related to pessimism. They carried out an online study where participants precompleted the judgment bias task and were assessed for anxiety (GAD7) and depression (PHQ9) as well as dispositional pessimism. The analyzed overall bias on the task and fit decisions to a drift diffusion model. They did not find any relationship with pessimism either with overall bias or with any parameters of the computational model the model. They did, however, show that anxiety and depression are related to lower drift rate and higher threshold. Overall, the paper is well written, and the topic is interesting. There are some areas of the analysis that are unclear and need to be clarified or improved as well as some conceptual questions**.

Again, we thank this reviewer for their positive comments on the manuscript, as well as their helpful suggestions below.

**Major comments:**

**1. The task used here seems very similar to the probabilistic reward task developed by Pizzagalli. It’s unclear why the literature on this task, which includes several papers using drift diffusion modeling, has not been incorporated into the introduction or discussion**.

## Author’s response

We thank the reviewer for highlighting the similarity between our task and Pizzagalli et al.’s probabilistic reward task (PRT). We have added an additional paragraph to the discussion section to explore similarities and differences between the two tasks, and how computational modelling can be applied to both to explore potential overlap of underlying cognitive mechanisms related to negative affective biases:

Lines 480-499:

“It is important to note that many other tasks have been developed to explore affective biases in decision-making. For example, the judgement bias task used here shares similarities with the probabilistic reward task (PRT) developed by Pizzagalli and colleagues, which has been widely used to study anhedonia and depression (Pizzagalli et al., 2008a; Pizzagalli et al., 2008b). In contrast to our high/low reward design, the PRT uses two stimuli reinforced with equal reward magnitude but differing probabilities, such that correct identification of one (‘rich’) stimulus is rewarded more frequently than the other (‘lean’) stimulus, leading healthy participants to develop a response bias towards the rich stimulus; this bias is typically reduced in individuals with anhedonia and depression (Pizzagalli et al., 2008a; Pizzagalli et al., 2008b). Drift-diffusion modelling of PRT performance has shown that this reduced response bias is associated with both reduced drift rate and reduced starting point bias (Shen et al., 2024). In contrast to judgement bias tasks, which rely on explicitly learned reward contingencies before testing responses to ambiguity, the PRT captures the gradual development of a response bias under uncertainty, requiring participants to learn reward probabilities over time (Pizzagalli et al., 2008a; Pizzagalli et al., 2008b). While some PRT studies link negative affect to altered learning rate, there is evidence to suggest that these effects instead reflect reduced reward sensitivity (Huys et al., 2013). In this case, the observed effects of negative affect on decision-making in both tasks may be driven by overlapping underlying mechanisms. The use of multiple complementary tasks such as these in the same participants, coupled with computational modelling of the data, would be beneficial in further characterising affective biases.”

## Reviewer B:

**2. The conclusion mostly restates the results. More interpretation and more connections to related literature would strengthen the paper**.

## Author’s response

We assume that the referee is referring to the Discussion which we believe does interpret the results in some depth, and provides context by comparing them to related literature. We consider a range of interpretational issues including whether and how the judgement bias task reflects a LOT-R type ‘optimism’ construct, links between task performance and questionnaire-based anxiety and depression constructs, potential influence of within-task changes on affective states, and implications of HDDM findings. We have now added discussion of the similar probabilistic-reward task, and interpretation of the effect sizes and potential influence of task order. Throughout, our discussion includes comparison to findings from relevant human and non-human animal studies, and suggestions for future work.

## Reviewer B:

**3. GAD7 and PHQ9 are highly correlated; therefore, it doesn’t make sense to first test a combined analysis where these are both in the regression. It’s unclear what this combined GLM is testing since any shared variance will not be included in the effects of the questionnaires**.

## Author’s response

We agree that given the strong correlation observed between anxiety (GAD-7) and depression (PHQ-8) scores, that these variables are not well-suited to be included in the same GLM if the aim is to interpret their unique effects. However, the initial combined GLM was included because this analysis was preregistered (https://osf.io/shvfr) and planned prior to data collection, at which point whether and to what extent the questionnaire scores would correlate with each other was unknown. Therefore, it was decided to run an initial GLM containing the following independent variables: age, sex, anxiety score (from the GAD-7), depression score (from the PHQ-8) and pessimism score (from the LOT-RP) to determine whether demographic and questionnaire variables were significantly associated with the proportion of ‘optimistic’ responses to ambiguous stimuli in the judgement bias task (Optimistic Responses).

In our preregistration we also anticipated that the three questionnaire scores would be highly correlated and therefore would require separate GLMs to independently test for associations between each questionnaire score and Optimistic Responses. Pearson’s correlations were used to assess the association between the three questionnaire scores and as these were highly correlated with each other as expected, we ran the three further GLMs, each containing one of the questionnaires as well as age and sex.

## Reviewer B:

**4. Relatedly, the GAD7 and PHQ9 do not measure separate constructs. If the authors want to measure anxiety versus depression, it would be better to use questionnaires developed for this, such as those based on the tripartite model of anxiety and depression**.

## Author’s response

We agree that as anxiety and depression share substantial variance, use of questionnaires based on the tripartite model of anxiety and depression, such as the Mood and Anxiety Symptom Questionnaire (MASQ), could be well suited to disentangling the relationships between anxiety, depression, and judgement bias. In the present study, the GAD-7 and PHQ-8 were selected as our aim was to capture negative affect in a manner that was both clinically relevant and comparable to existing literature. Although correlated, the GAD-7 and PHQ-8 were developed and have been validated as measures of generalised anxiety and depression respectively. Both are routinely used clinically in the UK to assess and monitor anxiety and depression symptom severity. Combining questionnaires based on the tripartite model with the judgement bias task offers an interesting direction for future research and we have updated our discussion section to mention this:

Lines 517-522: “Whilst it is possible that negative judgement bias is driven by a negative trait factor common to both anxiety and depression, future research could use more specific questionnaires, such as measures based on the tripartite model (e.g. the Mood and Anxiety Symptom Questionnaire; MASQ; Watson et al., 1995), to better dissociate anxiety and depression symptoms and explore whether particular symptoms or sets of symptoms are more strongly associated with judgement bias.”

## Reviewer B:

**5. In table 1 it’s unclear where the AIC values are coming from. Are these from the regression or from the DDM? It’s also unclear why these numbers vary so widely with different drift diffusion model parameters**.

## Author’s response

The AIC values in table one relate to the general linear models (GLMs) used to investigate associations between the questionnaire measures (self-reported anxiety, depression, and pessimism) and the drift-diffusion model parameters (drift rate, threshold separation, start point bias, and non-decision time). For each DDM parameter we ran a GLM containing age and sex, and three further GLMs containing age, sex, and one of the questionnaire scores: anxiety (GAD-7) score, depression (PHQ-8) score, or pessimism (LOT-RP) score. For each DDM parameter, the model with the best fit was selected using the lowest AIC value and results for this model only are reported.

The AIC values vary between DDM parameters as the GLMs are fit to different outcome variables which vary in size and range, for example our drift rate values range between -0.938 and 2.569, whereas the start point bias values range between 0.451 and 0.633. Consequently, AIC values are not comparable across models containing different DDM parameters. Here the models containing each DDM parameter were compared with each other to select the model which provided the best fit.

The title and legend for Table 1 have been updated to improve clarity:

“**Table 1. Model comparison for general linear models (GLMs) pairing drift-diffusion model (DDM) parameters with questionnaire predictors.** Akaike Information Criterion (AIC) values were used to compare model fit. The GLM with the lowest AIC value for each DDM parameter is highlighted and results for this model are reported.”

## Reviewer B:

**6. It appears that DDM parameters are first extracted from the model for each person and then analyzed in a regression. The software used here should have the ability to analyze the effects of questionnaires in the model itself, rather than having to carry out a two-stage analysis that decreases power**.

## Author’s response

While HDDM allows covariates to be incorporated directly into a DDM, our analysis used hierarchically estimated participant-level parameters rather than independent per-subject fits, such that individual estimates were regularised by group-level priors. Our choice was motivated by both statistical and interpretive considerations. Anxiety, depression, and pessimism questionnaire measures were correlated with each other, making their simultaneous inclusion as covariates within a single HDDM prone to multicollinearity and reduced parameter identifiability. These measures were therefore examined in separate models, which was more straightforward in a two-stage framework. We acknowledge that integrated one-stage models may offer greater power and results should be interpreted as associations between hierarchically estimated latent decision parameters and questionnaire measures.

We have updated the drift-diffusion modelling methods section to clarify this:

Lines 299-305: “DDM parameters were estimated using a fully hierarchical Bayesian model implemented in HDDM, such that individual estimates were regularised by group-level priors. Although HDDM allows covariates to be included directly, initial analysis revealed that anxiety, depression, and pessimism questionnaire scores were correlated, making their simultaneous inclusion prone to multicollinearity and reduced identifiability. Instead, relationships between DDM parameters and demographic and questionnaire variables were subsequently examined in separate general linear models (GLMs).”

## Reviewer B:

**7. Model checking for the drift diffusion model is needed – such as posterior predictive checks and parameter recovery**.

## Author’s response

We have now added more information on model checking to the drift-diffusion modelling methods section:

Lines 289-298: “We ran 10,000 MCMC iterations, discarding the first 500 samples as burn-in. Starting values were optimised prior to sampling to facilitate convergence. Model convergence was assessed through visual inspection of trace plots and autocorrelation functions. These diagnostics indicated stable post burn in sampling, good mixing, and no evidence of systematic trends. In addition, posterior predictive checks indicated that the fitted model adequately reproduced the observed reaction time distributions, suggesting satisfactory convergence and model fit. Parameter recovery checks showed good recovery, and a hold-out validation procedure confirmed good predictive performance for held-out data (see supplementary material for details).”

Further information on parameter recovery is included in the supplementary material (see response to reviewer 1’s third comment), and the code and associated plots for the original model (including posterior predictive plots) and parameter recovery are available here: https://doi.org/10.17605/OSF.IO/5JH6G

## Reviewer B:

**Minor:**

**8. Please state the correlation between the GAD7 and PHQ9 in the results or methods rather than in the discussion**.

## Author’s response

The correlation between anxiety (GAD-7) and depression (PHQ-8) has now been moved from the discussion to the results section:

Lines 333-335: “However, as the 3 questionnaires scores were highly correlated (particularly anxiety and depression: r(514)=.81, p < 0.001, Figure 5), we also ran separate GLMs for each of the questionnaires which controlled for sex and age.”

## Reviewer B:

**9. The discussion states that there is no evidence for a relationship between pessimism and performance on this task; however, this is not correct. Instead, the authors could not reject the null hypothesis that there is no relationship. The authors could use Bayesian analyses if they wanted to be able to show that there was no relationship**.

## Author’s response

We have updated our wording to reflect this in the abstract:

Lines 19-21: “However, we did not find sufficient evidence to reject the null hypothesis that self-reported dispositional pessimism is associated with ‘pessimistic’ responses in the task.”

And discussion:

Lines 437-440: “We also showed that while self-reported dispositional pessimism (LOT-RP) scores are significantly positively correlated with both anxiety and depression scores, we did not find sufficient evidence to reject the null hypothesis that self-reported pessimism is inversely associated with ‘optimistic’ responses in the judgement bias task.”

Lines 609-611: “However, contrary to our hypothesis, our results did not provide sufficient evidence for an association between self-reported dispositional pessimism and ‘pessimistic’ responses in the judgement bias task.”

***All new and updated code has been uploaded/reuploaded to the OSF and is available here: https://doi.org/10.17605/OSF.IO/5JH6G***

# Reviewer report: Round 2, Author Response

**DOI:** 10.5334/cpsy.167.r6

Dear Dr Dongil Chung,

Ref: The Effects of Negative Affect and Dispositional Pessimism on Decision-Making Under Ambiguity

We thank you and the reviewers for your comments on our revised manuscript. We have addressed the minor requested revision and provide details of this below. We hope that our work is now suitable for publication.

Yours sincerely,

Molly Davidson, Vikki Neville, John Fennell, Elizabeth Paul, Iain Gilchrist, Michael Mendl

## Reviewer B:

**Reviewer 2 minor revision:**

**The high correlation between GAD-7 and PHQ-8 scores… the authors acknowledge negligible differences in model fit (AIC difference < 1) yet frame anxiety as the “better predictor” in their initial model. Their solution is to reframe the finding as measuring a single “negative affect” construct rather than distinguishing anxiety from depression. While this reframing is reasonable and now clearly stated in the discussion, it does not resolve the original limitation. The reader should understand that the authors cannot determine whether anxiety, depression, or their shared variance is driving the observed effect. I would recommend the discussion make this limitation even more explicit as well as rewording this result in the paper overall – e.g. that given the high multicollinearity between anxiety and depression measures, one cannot distinguish which is the primary driver and that the findings indicate negative affect more broadly influences decision-making under ambiguity**.

## Author’s response

We agree with the reviewer and recognise the limitation of multicollinearity between our anxiety and depression measures when aiming to explore whether anxiety or depression symptoms drive ‘pessimistic’ decision-making in the judgement bias task. We have updated the wording of our interpretation in the discussion to emphasise this (see below), and in the same paragraph offer suggestions for future studies to use different questionnaires which may better dissociate anxiety and depression symptoms.

Lines 504-507: “Given the high multicollinearity between anxiety and depression measures, it is not possible to determine which is the primary driver of our observed results. Accordingly, we suggest that the findings indicate negative affect more broadly influences ‘pessimistic’ decision-making under ambiguity.”
